# iTRAQ Analysis of Complex Proteome Alterations in 3xTgAD Alzheimer's Mice: Understanding the Interface between Physiology and Disease

**DOI:** 10.1371/journal.pone.0002750

**Published:** 2008-07-23

**Authors:** Bronwen Martin, Randall Brenneman, Kevin G. Becker, Marjan Gucek, Robert N. Cole, Stuart Maudsley

**Affiliations:** 1 Laboratory of Neurosciences, National Institute on Aging Intramural Research Program, Biomedical Research Center, Baltimore, Maryland, United States of America; 2 Research Resources Branch, National Institute on Aging Intramural Research Program, Biomedical Research Center, Baltimore, Maryland, United States of America; 3 Mass Spectrometry/Proteomics Facility at Johns Hopkins School of Medicine, Baltimore, Maryland, United States of America; Swiss Federal Institute of Technology Lausanne, Switzerland

## Abstract

Alzheimer's disease (AD) is characterized by progressive cognitive impairment associated with accumulation of amyloid β-peptide, synaptic degeneration and the death of neurons in the hippocampus, and temporal, parietal and frontal lobes of the cerebral cortex. Analysis of postmortem brain tissue from AD patients can provide information on molecular alterations present at the end of the disease process, but cannot discriminate between changes that are specifically involved in AD versus those that are simply a consequence of neuronal degeneration. Animal models of AD provide the opportunity to elucidate the molecular changes that occur in brain cells as the disease process is initiated and progresses. To this end, we used the 3xTgAD mouse model of AD to gain insight into the complex alterations in proteins that occur in the hippocampus and cortex in AD. The 3xTgAD mice express mutant presenilin-1, amyloid precursor protein and tau, and exhibit AD-like amyloid and tau pathology in the hippocampus and cortex, and associated cognitive impairment. Using the iTRAQ stable-isotope-based quantitative proteomic technique, we performed an in-depth proteomic analysis of hippocampal and cortical tissue from 16 month old 3xTgAD and non-transgenic control mice. We found that the most important groups of significantly altered proteins included those involved in synaptic plasticity, neurite outgrowth and microtubule dynamics. Our findings have elucidated some of the complex proteome changes that occur in a mouse model of AD, which could potentially illuminate novel therapeutic avenues for the treatment of AD and other neurodegenerative disorders.

## Introduction

Alzheimer's disease (AD) is a progressive neurodegenerative disorder which affects mainly the elderly population and it is the most common form of dementia and cognitive impairment [1]. Considerable progress has been made in recent years towards understanding the pathogenesis and underlying mechanisms of AD. AD is characterized by widespread neurodegeneration throughout the association cortex, limbic system and hippocampus. Alterations in the processing of amyloid precursor protein (APP), resulting in the accumulation of amyloid β-peptide (Aβ) and the formation of oligomers, leads to synaptic damage and neurodegeneration. Deposition of Aβ also occurs in the neuropil and around the blood vessels, and has been shown to result in the formation of neurofibrillary tangles [2]–[4]. Accompanying the neuronal damage, there is activation of macrophage/microglial cells and astroglial cells that produce specific cytokines and chemokines. In the initial stages of AD, the neurodegenerative process may target the synaptic terminals [5], [6] and then propagate to axons and dendrites, leading to neuronal dysfunction and eventually to neuronal death [7]. Neurofibrillary tangle formation with accumulation of phosphorylated tau is also an important pathologic process in AD and has been linked to the cognitive alterations in these patients [8].

The neurodegenerative process in AD is thought to initiate in the entorhinal cortex and then disseminates into the hippocampus [9] and neocortical regions in the temporal, parietal and frontal lobes [10]. The perforant pathway connects neurons in the entorhinal cortex layer 2 with the hippocampal dentate gyrus and is considered to play an important role in early memory formation [11]. The most significant correlate to the severity of the cognitive impairment in AD is the loss of synapses in the frontal cortex and limbic system [12]. This pathogenic process involves changes in synaptic plasticity that includes alterations in the formation of synaptic contacts, changes in dendritic spine morphology and abnormal synaptic contact [13]. However, other cellular mechanisms necessary to maintain synaptic plasticity may also be affected in AD [5], [14]. While the molecular alterations that result in synaptic dysfunction in AD are presently unclear, oxidative stress, perturbed cellular energy metabolism and calcium homeostasis have been implicated [15].

Considerable progress has been made in recent years towards gaining a better understanding of the pathogenesis of AD, and in developing novel therapeutic approaches. In this regard, the identification of gene mutations that cause inherited forms of AD, and the generation of transgenic mouse models of AD that express the mutant human genes have been invaluable. Studies of mice expressing APP, presenilin-1 (PS-1) and/or tau mutations have demonstrated the potential of strategies that reduce Aβ accumulation, increase Aβ clearance, and reduce oxidative damage [16]. However, there are likely to be many additional factors involved in AD pathogenesis that, once fully elucidated and understood, may also provide novel approaches for preventing and treating AD. In this study, we used the well characterized mass-tag labeling proteomic technique, iTRAQ, to elucidate some of the complex proteome changes that occur in a mouse model of AD. Using 3xTgAD mice that express mutant PS-1, mutant APP and mutant tau [17], we performed a detailed proteomic analysis of a large number of quantitatively differentially expressed proteins in the hippocampal and cortical tissues of 16-month old 3xTgAD mice compared to age-matched non-transgenic controls. Proteins significantly altered in the 3xTgAD mice compared to controls included those involved in synaptic plasticity, neurite outgrowth and microtubule dynamics.

## Results

### Measurement of relative protein levels between control and AD mice

**Figure 1:**
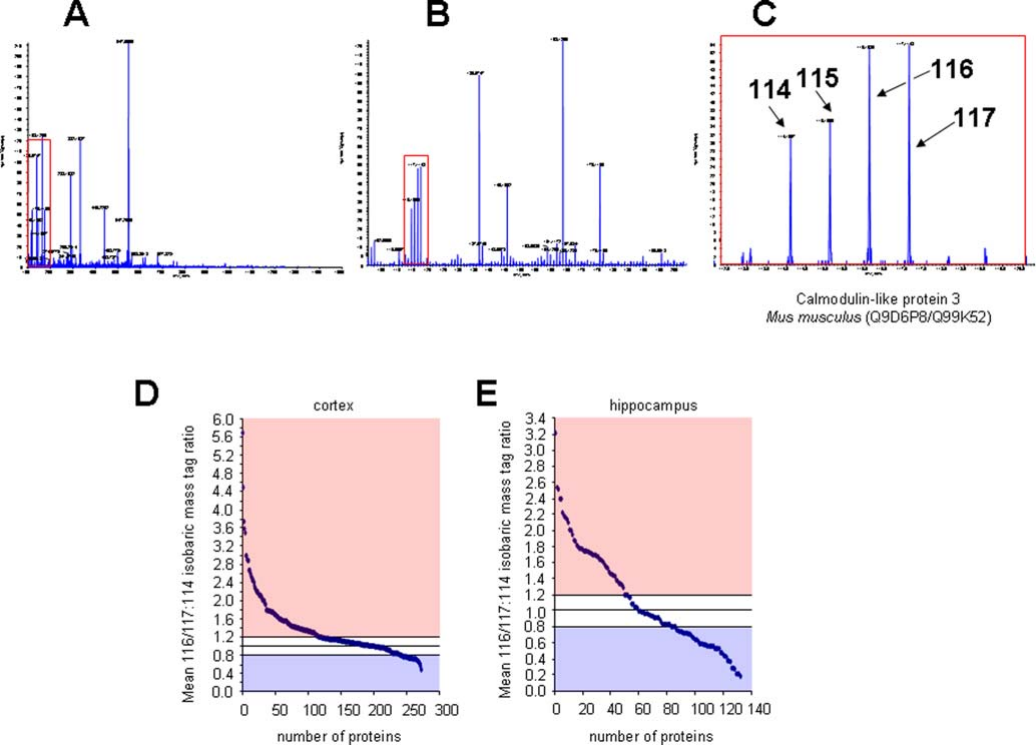
Sample iTRAQ analysis and iTRAQ ratios (3xTgAD:control) for cortex and hippocampus. A–C. Semi-quantitative isobaric mass-tag identification from a single MS/MS peptide collision-induced dissociation event. A. A full scan MS/MS event for a single identified parent ion with its multiple b and y series daughter ions shown. The isobaric mass tag labels added were 114 and 115 for control samples and 116 and 117 for 3xTgAD samples. The red box displayed upon the MS/MS spectra is successively expanded in panels B and C to identify the low mass range end of the MS/MS scan event, exposing the mass range up to 120 m/z. In this example the 3xTgAD samples yielded the greater overall amount of isobaric mass tag shown by the greater peak volumes for the 116- and 117-labeled samples compared to the 114- and 115-labelled samples. D and E. iTRAQ ratios of 3xTgAD versus control for cortex and hippocampus. D. Mean 116/117:114/115 isobaric mass tag ratios for samples resolved by MS/MS from the cortex of both control and 3xTgAD mice. Proteins identified from peptides that displayed a ratio greater than or equal to 1.2 or less that or equal to 0.8 are considered to be statistically different according to standard protocols from unity and therefore distinctly regulated compared to control. E. Correlated data gathered from mixed control and 3xTgAD hippocampal samples.

Peptides generated from trypsin digestion of control and AD brain proteins were labeled at their free amine sites using the isobaric mass tag labels (control 114, 115: AD 116, 117), mixed together and analyzed by reverse phase liquid chromatography coupled to tandem mass spectrometry. Upon collision-induced dissociation, the parent peptides were broken up and the associated isobaric mass tags were released. The dissociation of the parent peptide yielded a characteristic mass fragmentation pattern (Fig. 1A–C) that enabled identification of the parent protein by comparing this fragmentation fingerprint to theoretical digests of proteins. Additionally the associated isobaric mass tags were released, allowing the measurement of the relative levels of the mass labels for each parent peptide from the two sample types (control and AD). Therefore, comparative peptide data between control and AD samples could be obtained for multiple proteins from one experiment. This process greatly reduces any variability of peptide measurement for control versus AD samples.

The relative levels for a single exemplar protein (calmodulin-like protein 3, Calml3, Q9D6P8/Q99K52) are shown in Fig. 1A–C, in which the AD-labeled mass tags (116, 117) were significantly higher than the levels of the same peptide from control animals (114, 115).

### Global alterations in protein expression level in the cortex and hippocampus of male control and AD animals

iTRAQ mass tag ratios were calculated for proteins with reliable identifications (see Materials and Methods) based upon their collision-induced dissociation fragmentation patterns. Ratios of the AD mass tags (116, 117) compared to the control tags (114, 115) that were greater than 1.2 or less than 0.8, were considered to be statistically different from unity, *i.e.* changed expression level of the protein. The numbers of up-regulated and down-regulated proteins are displayed in Figs. 1D and E. We found that generally more proteins were detected in the cortical samples than in the hippocampal samples. The specific proteins and their expression alterations (fold change compared to control mass tag levels) are listed for the cortex and hippocampus in Tables 1 and 2, respectively.

**Table 1:** Fold change of protein level in the cerebral cortex of 16 month old male 3xTgAD mice compared to age-matched controls.

ID	Cortex protein	
Q5NC92_MOUSE (Q5NC92)	Novel histone H2A family member	5.7
Q8R1M2_MOUSE (Q8R1M2)	H2A histone family member J	4.5
Q6PDS7 (Q6PDS7)	Histone 1h4h	3.75
H2A1_MOUSE (P22752)	Histone H2A.1	3.59
PEP19_MOUSE (P63054)	PEP-19	3.5
Q9D9Y2_MOUSE (Q9D9Y2)	Adult male testis cDNA: tubulin alpha 7	3
VDAC1_MOUSE (Q60932)	Voltage-dependent anion-selective anion channel 1 (VDAC1)	3
ATPD_MOUSE (Q9D3D9)	ATP synthase delta	2.9
GSTP1_MOUSE (P19157)	Glutathione S-transferase P1	2.88
BASP_MOUSE (Q91XV3)	Brain acid soluble protein 1 (BASP1 protein) (Neuronal axonal membrane protein NAP-22)	2.88
Q71B07 (Q71B07)	Transmembrane protein	2.67
SNP25_MOUSE (P60879)	Synaptosomal-associated protein 25 (SNAP-25) (Synaptosomal-associated 25 kDa protein)	2.66
CYC2_MOUSE (P00015)	Cytochrome c	2.6
AMPH_MOUSE (Q7TQF7)	Amphiphysin	2.55
SYUA_MOUSE (O55042)	Alpha-synuclein (Non-A beta component of AD amyloid) (Non-A4 component of amyloid precursor) (NACP)	2.5
Q6P5D0 (Q6P5D0)	Dihydropyriminidase like-2	2.46
1433Z_MOUSE (P63101)	14-3-3 protein zeta/delta (Protein kinase C inhibitor protein 1) (KCIP-1) (SEZ-2)	2.45
ATP5E_MOUSE (P56382)	ATP synthase epsilon	2.4
PKP3_MOUSE (Q9QY23)	Plakophilin	2.33
Q8CEV2_MOUSE (Q8CEV2)	12 days embryo head cDNA: gyceraldehyde-3-phosphate dehydrogenase homolog	2.27
Q8VCE0 (Q8VCE0)	Na+/K+ ATPase alpha 3 subunit	2.25
G3P_MOUSE (P16858)	GAPDH	2.2
Q3TUZ9 (Q3TUZ9)	Adult male tongue	2.2
TBA1_MOUSE (P68369)	Alpha 1 tubulin	2.2
SYN1_MOUSE (O88935)	Synapsin	2.15
ATP4A_MOUSE (Q64436)	K+ transporting ATPase chain 1	2.14
STXB1_MOUSE (O08599)	Syntaxin-binding protein	2.12
TBA4_MOUSE (P68368)	Tubulin alpha4 chain	2.11
DYL2_MOUSE (Q9D0M5)	Dynein	2.11
PPIA_MOUSE (P17742)	Peptidylprolyl cistransisomerase A	2.08
Q9CXK3_MOUSE (Q9CXK3)	14,17 days embryo head cDNA: alpha actin	2.03
CYC_MOUSE (P62897)	Cytochrome c somatic	2
SNAB_MOUSE (P28663)	Beta soluble NSF	2
TBA2_MOUSE (P05213)	Alpha tubulin	2
CLCB_MOUSE (Q6IRU5)	Light chain clathrin	1.94
HBAZ_MOUSE (P06467)	Hemoglobin zeta subunit	1.92
Q4VBD1_MOUSE (Q4VBD1)	Hypothetical protein AK190093	1.8
Q6ZQ49_MOUSE (Q6ZQ49)	MKIAA0778	1.8
MAP2_MOUSE (P20357)	MAP-2	1.8
Q3U452 (Q3U452)	NOD-derived CD11+ve dendritic cells	1.78
STMN2_MOUSE (P55821)	Stathmin-2	1.78
KCRB_MOUSE (Q04447)	Creatine kinase B	1.77
COF2_MOUSE (P45591)	Coflin	1.77
TPM3_MOUSE (P21107)	Tropomyosin alpha 3 chain	1.75
HXK1_MOUSE (P17710)	Hexokinase	1.75
AT1A4_MOUSE (Q9WV27)	Na+/K+ ATPase chain 4	1.75
SYPH_MOUSE (Q62277)	Synaptophysin (Major synaptic vesicle protein p38) (BM89 antigen)	1.75
1433E_MOUSE (P62259)	14-3-3 epsilon	1.74
Q8BWN0_MOUSE (Q8BWN0)	Adult pancreas islet cell cDNA: tyrosine 3-monooxygenase	1.7
Q922A0 (Q922A0)	Eno2	1.7
AT1A2_MOUSE (Q6PIE5)	Na+/K+ transporting ATPase	1.7
ALDOA_MOUSE (P05064)	Fructose bisphosphate aldolase A	1.66
ARL11_MOUSE (Q6P3A9)	ARF-like 11	1.66
GNAO2_MOUSE (P18873)	Go alpha 2	1.65
ENOA_MOUSE (P17182)	Alpha enolase	1.64
Q5M9J7 (Q5M9J7)	NADH dehydrogenase (Ubiquinone) Fe-S protein 6	1.62
STMN1_MOUSE (P54227)	Stathmin	1.6
ARF6_MOUSE (P62331)	ARF6	1.6
TPM2_MOUSE (P58774)	Tropomyosin beta chain (Tropomyosin 2)	1.59
GBG2_MOUSE (P63213)	Guanine nucleotide-binding protein G(I)/G(S)/G(O) gamma-2 subunit (G gamma-I)	1.58
LATS1_MOUSE (Q8BYR2)	Serin/threonine-protein kinase LATS1	1.57
Q3U764 (Q3U764)	Bone marrow macrophage cDNA: heat shock protein 8	1.56
PGK1_MOUSE (P09411)	Phosphoglycerate kinase 1 (EC 2.7.2.3)	1.555
TBB3_MOUSE (Q9ERD7)	Tubulin beta3 chain	1.55
Q8JZW6 (Q8JZW6)	Rpgr protein	1.55
Q8C5K4 (Q8C5K4)	Adult male olfactory brain cDNA: microtubule associated protein tau	1.55
GNA12_MOUSE (P27600)	G protein Xla	1.55
O55129 (O55129)	STOP protein	1.55
Q3TUI2 (Q3TUI2)	Protein BAT4	1.5
Q99K52 (Q99K52)	Calmodulin-like 3	1.5
COX6C_MOUSE (Q9CPQ1)	Cytochrome c oxidase polypeptide Vic	1.5
SODC_MOUSE (P08228)	Superoxide dismutase	1.48
Q91ZZ3 (Q91ZZ3)	Beta synuclein	1.46
HBB1_MOUSE (P02088)	Hemoglobin beta 1 subunit	1.45
FKB1A_MOUSE (P26883)	Rotamase	1.45
P25A_MOUSE (Q7TQD2)	Tubulin polymerization protein (TPPP)	1.44
Q9R0S6 (Q9R0S6)	Beta-1-globin (Fragment)	1.44
ARF5_MOUSE (P84084)	ADP-Ribosylation factor 5	1.43
Q9D2U9 (Q9D2U9)	Adult male cerebellum cDNA: histone H2B	1.43
MBP_MOUSE (P04370)	Myelin basic protein	1.42
HBA_MOUSE (P01942)	Hemoglobin alpha subunit	1.42
Q3TBV8 (Q3TBV8)	NOD-derived CD11+ve dendritic cell cDNA: pyruvate kinase	1.41
KCRU_MOUSE (P30275)	Creatine kinase, ubiquitous mitochondrial precursor (EC 2.7.3.2) (U-MtCK) (Mia-CK)	1.41
Q8VC46 (Q8VC46)	Cortex ubc protein	1.4
Q9DD04 (Q9DD04)	Adult male kidney cDNA clone 061: ADP ribosylation factor 4	1.4
VATE_MOUSE (P50518)	Vaculoar ATP synthase subunit E	1.4
HBB2_MOUSE (P02089)	Hemoglobin beta 2 subunit	1.39
TBB6_MOUSE (Q922F4)	Tubulin beta chain	1.38
Q9CWF2 (Q9CWF2)	ES cells cDNA	1.38
Q7TMM9 (Q7TMM9)	Tubulin beta 2	1.38
FUS_MOUSE (P56959)	RNA-binding protein FUS (Pigpen protein)	1.38
Q3UH19 (Q3UH19)	Adult male brain cDNA RIKEN: microtubule associated protein tau	1.36
CLCA_MOUSE (O08585)	Clathrin	1.36
TPIS_MOUSE (P17751)	Triosephosphate isomerase	1.36
STIP1_MOUSE (Q60864)	Stress-induced-phosphoprotein 1 (STI1) (Hsc70/Hsp90-organizing protein) (Hop) (mSTI1)	1.355
AT12A_MOUSE (Q9Z1W8)	K+ transporting ATPase	1.35
TAU_MOUSE (P10637)	Microtubule-associated protein tau (Neurofibrillary tangle protein) (Paired helical filament-tau)	1.35
Q9QZ83 (Q9QZ83)	Gamma actin-like protein	1.34
CPLX2_MOUSE (P84086)	Complexin-2 (Synaphin-1) (921-L)	1.34
ATPA_MOUSE (Q03265)	ATP synthase alpha chain, mitochondrial precursor (EC 3.6.3.14)	1.34
AN32A_MOUSE (O35381)	Acidic leucine-rich nuclear phosphoprotein	1.33
ACTG_MOUSE (P63260)	Cytoplasmic actin	1.33
ALBU_MOUSE (P07724)	Serum albumin precursor	1.33
TBA6_MOUSE (P68373)	Tubulin alpha-6 chain (Alpha-tubulin 6) (Alpha-tubulin isotype M-alpha-6)	1.32
Q9CY10 (Q9CY10)	13 days embryo liver cDNA, RIKEN full-length enriched library, clone:2510040B16 product:hemoglobin beta major chain	1.315
Q5XJF8 (Q5XJF8)	Tubulin, alpha 1	1.305
TBBX_MOUSE (P68372)	Beta tubulin	1.3
CXCC1_MOUSE (Q9CWW7)	CpG binding protein	1.3
Q6W8Q3 (Q6W8Q3)	Purkinje cell protein 4 like-1	1.29
CISY_MOUSE (Q9CZU6)	Citrate synthase, mitochondrial precursor (EC 2.3.3.1)	1.29
UCRH_MOUSE (P99028)	Ubiquinol-cytochrome c reductase complex 11 kDa protein, mitochondrial precursor (EC 1.10.2.2)	1.29
ATP5J_MOUSE (P97450)	ATPsynthase coupling factor 6	1.25
UCRQ_MOUSE (Q9CQ69)	Ubuiqinol 1 cytochrome c reductase complex	1.24
ROA3_MOUSE (Q8BG05)	Heterogenous nuclear riboprotein A3	1.235
Q9R0S6 (Q9R0S6)	Beta 1 globin	1.23
Q5D0E8 (Q5D0E8)	Hbb-B1 protein	1.22
ABCG1_MOUSE (Q64343)	ATP binding cassette subfamily G member	1.2
MT3_MOUSE (P28184)	Metallothionein-3 (MT-3) (Metallothionein-III) (MT-III) (Growth inhibitory factor) (GIF)	−1.2
Q3V2G3 (Q3V2G3)	Adult male small intestine cDNA, RIKEN full-length enriched library, clone:2010010O17 product:Trypsinogen 16 homolog	−1.22
STX1C_MOUSE (P61264)	Syntaxin-1B2 (Syntaxin 1B)	−1.24
PGAM1_MOUSE (Q9DBJ1)	Phosphoglycerate mutase 1 (EC 5.4.2.1) (EC 5.4.2.4) (EC 3.1.3.13) (Phosphoglycerate mutase isozyme B)	−1.25
ENOG_MOUSE (P17183)	Gamma-enolase (EC 4.2.1.11) (2-phospho-D-glycerate hydro-lyase) (Neural enolase)	−1.27
THIO_MOUSE (P10639)	Thioredoxin (ATL-derived factor) (ADF)	−1.27
Q3TVQ0 (Q3TVQ0)	Osteoclast-like cell cDNA, RIKEN full-length enriched library, clone:I420044H02 product:formyltetrahydrofolate synthetase domain containing 1	−1.28
Q8BMV6 (Q8BMV6)	0 day neonate eyeball cDNA, RIKEN full-length enriched library, clone:E130116L18 product:hypothetical Type I antifreeze protein	−1.29
Q4VWZ5 (Q4VWZ5)	Diazepam binding inhibitor, splice form 1b	−1.3
HARP_MOUSE (Q8K3X6)	Harmonin-interacting ankyrin-repeat containing protein (Harp)	−1.3
ATPB_MOUSE (P56480)	ATP synthase beta chain, mitochondrial precursor (EC 3.6.3.14)	−1.31
ENSA_MOUSE (P60840)	Alpha-endosulfine	−1.34
Q505F3 (Q505F3)	Minichromosome maintenance protein 10	−1.35
VAMP2_MOUSE (P63044)	Vesicle-associated membrane protein 2 (VAMP-2) (Synaptobrevin-2)	−1.35
TOP2B_MOUSE (Q64511)	DNA topoisomerase 2-beta (EC 5.99.1.3) (DNA topoisomerase II, beta isozyme)	−1.35
CX7A2_MOUSE (P48771)	Cytochrome c oxidase polypeptide VIIa-liver/heart, mitochondrial precursor (EC 1.9.3.1)	−1.38
ATOX1_MOUSE (O08997)	Copper transport protein ATOX1 (Metal transport protein ATX1)	−1.38
Q9CRC1 (Q9CRC1)	Adult male testis cDNA, RIKEN full-length enriched library, clone:4933425L11 product:fructose-bisphosphate aldolase A	−1.4
Q80ZL4 (Q80ZL4)	Mtap2 protein	−1.4
Q4VWZ5 (Q4VWZ5)	Diazepam binding inhibitor, splice form 1b	−1.42
Q80X68 (Q80X68)	Citrate synthase-like protein (Adult male testis cDNA, RIKEN full-length enriched library, clone:492	−1.43
GPM6B_MOUSE (P35803)	Neuronal membrane glycoprotein M6-b (M6b)	−1.5
SPA4L_MOUSE (Q9DA32)	Sperm-associated antigen 4-like protein	−1.52
Q78PG9 (Q78PG9)	Coiled-coil domain-containing protein 25	−1.52
Q8C2C1 (Q8C2C1)	2 days neonate thymus thymic cells cDNA, RIKEN full-length enriched library, clone:E430030L01 product: hypothetical protein	−1.6
PRVA_MOUSE (P32848)	Parvalbumin alpha	−1.7
UBIQ_MOUSE (P62991)	Ubiquitin	−1.8
VAMP3_MOUSE (P63024)	Vesicle-associated membrane protein 3 (VAMP-3) (Synaptobrevin-3) (Cellubrevin) (CEB)	−2.4
Q6PHZ2 (Q6PHZ2)	Calcium/calmodulin-dependent kinase II	−2.5
VKORL_MOUSE (Q6TEK5)	Vitamin K epoxide reductase complex subunit 1-like protein 1 (VKORC1-like protein 1)	−3.4
FBRL_MOUSE (P35550)	Fibrillarin (Nucleolar protein 1)	−3.4

**Table 2:** Fold change of protein level in the hippocampus of 16 month old male 3xTgAD mice compared to age-matched controls.

ID	Hippocampal protein	
AMPH_MOUSE (Q7TQF7)	Amphiphysin	3.22
MBP_MOUSE (P04370)	Myelin basic protein (MBP) (Myelin A1 protein)	2.54
TCTP_MOUSE (P63028)	Translationally-controlled tumor protein (TCTP) (p23) (21 kDa polypeptide) (p21)	2.52
HBE_MOUSE (P02104)	Hemoglobin epsilon-Y2 subunit (Hemoglobin epsilon-Y2 chain) (Epsilon-Y2-globin)	2.4
HBB2_MOUSE (P02089)	Hemoglobin beta-2 subunit (Hemoglobin beta-2 chain) (Beta-2-globin) (Hemoglobin beta-minor chain)	2.4
Q9R0S6 (Q9R0S6)	Beta-1-globin (Fragment)	2.225
Q8R5L1 (Q8R5L1)	P32-RACK (Complement component 1, q subcomponent binding protein)	2.18
MAP2_MOUSE (P20357)	Microtubule-associated protein 2 (MAP 2)	2.16
Q91V86 (Q91V86)	11 days embryo whole body cDNA, RIKEN full-length enriched library, clone:2700082N11 product:hemoglobin beta	2.14
P25A_MOUSE (Q7TQD2)	Tubulin polymerization-promoting protein (TPPP)	2.1
CPLX2_MOUSE (P84086)	Complexin-2 (Synaphin-1) (921-L)	2.01
STX1C_MOUSE (P61264)	Syntaxin-1B2 (Syntaxin 1B)	2
SYUA_MOUSE (O55042)	Alpha-synuclein (Non-A beta component of AD amyloid) (Non-A4 component of amyloid precursor) (NACP)	1.95
Q9JI95 (Q9JI95)	CPN10-like protein	1.88
CH10_MOUSE (Q64433)	10 kDa heat shock protein, mitochondrial (Hsp10) (10 kDa chaperonin) (CPN10)	1.87
Q921J3 (Q921J3)	Dnajc5 protein	1.82
S100B_MOUSE (P50114)	S-100 calcium-binding protein beta subunit (S-100 protein, beta chain)	1.8
PRVA_MOUSE (P32848)	Parvalbumin alpha	1.78
Q8K0Z5 (Q8K0Z5)	Tropomyosin 3, gamma	1.78
NUYM_MOUSE (Q9CXZ1)	NADH-ubiquinone oxidoreductase 18 kDa subunit, mitochondrial precursor (EC 1.6.5.3) (EC 1.6.99.3)	1.77
FUS_MOUSE (P56959)	RNA-binding protein FUS (Pigpen protein)	1.75
SYPH_MOUSE (Q62277)	Synaptophysin (Major synaptic vesicle protein p38) (BM89 antigen)	1.75
GBG2_MOUSE (P63213)	Guanine nucleotide-binding protein G(I)/G(S)/G(O) gamma-2 subunit (G gamma-I)	1.75
Q9D6X2 (Q9D6X2)	Adult male tongue cDNA, RIKEN full-length enriched library, clone:2310046N07 product:peroxiredoxin 6	1.74
PEA15_MOUSE (Q62048)	Astrocytic phosphoprotein PEA-15	1.73
ATP5H_MOUSE (Q9DCX2)	ATP synthase D chain, mitochondrial (EC 3.6.3.14)	1.72
PEBP_MOUSE (P70296)	Phosphatidylethanolamine-binding protein (PEBP) (HCNPpp)	1.706
HPCA_MOUSE (P84075)	Neuron-specific calcium-binding protein hippocalcin	1.7
HINT1_MOUSE (P70349)	Histidine triad nucleotide-binding protein 1 (Adenosine 5′-monophosphoramidase)	1.7
Q8BGY2 (Q8BGY2)	Eukaryotic translation factor 5A-2	1.68
NUPM_MOUSE (Q9DCJ5)	NADH-ubiquinone oxidoreductase 19 kDa subunit (EC 1.6.5.3) (EC 1.6.99.3) (Complex I-19KD) (CI-19KD)	1.67
VATE_MOUSE (P50518)	Vacuolar ATP synthase subunit E (EC 3.6.3.14) (V-ATPase E subunit) (Vacuolar proton pump E subunit)	1.64
O55107 (O55107)	Basigin (Fragment)	1.633
PRDX5_MOUSE (P99029)	Peroxiredoxin 5, mitochondrial precursor (EC 1.11.1.15) (Prx-V) (Peroxisomal antioxidant enzyme)	1.62
UBIQ_MOUSE (P62991)	Ubiquitin	1.6
Q9CX22 (Q9CX22)	12 days embryo embryonic body between diaphragm region and neck cDNA, RIKEN full-length enriched library Cofilin 1	1.56
1433E_MOUSE (P62259)	14-3-3 protein epsilon (14-3-3E)	1.54
Q8BP43 (Q8BP43)	8 days embryo whole body cDNA, RIKEN full-length enriched library, clone:5730544G20 product:tropomyosin 1 alpha	1.53
VISL1_MOUSE (P62761)	Visinin-like protein 1 (VILIP) (Neural visinin-like protein 1) (NVL-1) (NVP-1)	1.48
Q3V1V5 (Q3V1V5)	Adult male brain cDNA, RIKEN full-length enriched library, clone:3526403B12 product:Spectrin alpha c	1.46
Q6PKE6 (Q6PKE6)	Platelet-activating factor acetylhydrolase, isoform 1b, alpha2 subunit (Bone marrow macrophage cDNA)	1.45
Q8R4B4 (Q8R4B4)	Down syndrome cell adhesion molecule-like protein (Fragment)	1.44
AATM_MOUSE (P05202)	Aspartate aminotransferase, mitochondrial precursor (EC 2.6.1.1) (Transaminase A)	1.43
SH3G2_MOUSE (Q62420)	SH3-containing GRB2-like protein 2 (EC 2.3.1.-) (SH3 domain protein 2A) (Endophilin 1) (SH3p4)	1.38
SODC_MOUSE (P08228)	Superoxide dismutase [Cu-Zn] (EC 1.15.1.1)	1.371
NDKA_MOUSE (P15532)	Nucleoside diphosphate kinase A (EC 2.7.4.6) (NDK A) (NDP kinase A) (Tumor metastatic process-associ	1.34
Q3UK59 (Q3UK59)	CRL-1722 L5178Y-R cDNA, RIKEN full-length enriched library, clone:I730025H19 product:ATPase, H+ transporting VI ATPase, H+ transporting, V1 subunit E	1.34
NUFM_MOUSE (Q9CPP6)	NADH-ubiquinone oxidoreductase 13 kDa-B subunit (EC 1.6.5.3) (EC 1.6.99.3) (Complex I-13Kd-B)	1.315
TPIS_MOUSE (P17751)	Triosephosphate isomerase (EC 5.3.1.1) (TIM) (Triose-phosphate isomerase)	1.29
UCHL1_MOUSE (Q9R0P9)	Ubiquitin carboxyl-terminal hydrolase isozyme L1 (EC 3.4.19.12) (UCH-L1)	1.2
SNP25_MOUSE (P60879)	Synaptosomal-associated protein 25 (SNAP-25) (Synaptosomal-associated 25 kDa protein)	1.2
PAK3_MOUSE (Q61036)	Serine/threonine-protein kinase PAK 3 (EC 2.7.1.37) (p21-activated kinase 3) (PAK-3) (Beta-PAK)	−1.25
CAP1_MOUSE (P40124)	Adenylyl cyclase-associated protein 1 (CAP 1)	−1.3
EFTU_MOUSE (Q8BFR5)	Elongation factor Tu, mitochondrial precursor	−1.35
ATPG_MOUSE (Q91VR2)	ATP synthase gamma chain, mitochondrial precursor (EC 3.6.3.14)	−1.35
ENOA_MOUSE (P17182)	Alpha-enolase (EC 4.2.1.11) (2-phospho-D-glycerate hydro-lyase) (Non-neural enolase) (NNE)	−1.36
AT1B1_MOUSE (P14094)	Sodium/potassium-transporting ATPase beta-1 chain (Sodium/potassium-dependent ATPase beta-1 subunit)	−1.4
Q80ZZ7 (Q80ZZ7)	Ank2 protein (Fragment)	−1.4
CH60_MOUSE (P63038)	60 kDa heat shock protein, mitochondrial precursor (Hsp60) (60 kDa chaperonin) (CPN60)	−1.4
Q3U764 (Q3U764)	Bone marrow macrophage cDNA, RIKEN full-length enriched library, clone:I830086I04 product:heat shock protein 8	−1.4
SUCA_MOUSE (Q9WUM5)	Succinyl-CoA ligase [GDP-forming] alpha-chain, mitochondrial precursor (EC 6.2.1.4)	−1.4
TBB5_MOUSE (P99024)	Tubulin beta-5 chain	−1.43
DNM1L_MOUSE (Q8K1M6)	Dynamin-1-like protein (EC 3.6.5.5) (Dynamin-related protein 1) (Dynamin family member proline-rich)	−1.5
PAK1_MOUSE (O88643)	Serine/threonine-protein kinase PAK 1 (EC 2.7.1.37) (p21-activated kinase 1) (PAK-1) (P65-PAK)	−1.5
Q58ES7 (Q58ES7)	Spna2 protein (Fragment)	−1.51
CALX_MOUSE (P35564)	Calnexin precursor	−1.52
CRYM_MOUSE (O54983)	Mu-crystallin homolog	−1.53
Q6PCN2 (Q6PCN2)	Ankyrin 2, brain	−1.54
ATPB_MOUSE (P56480)	ATP synthase beta chain, mitochondrial precursor (EC 3.6.3.14)	−1.57
VATA1_MOUSE (P50516)	Vacuolar ATP synthase catalytic subunit A, ubiquitous isoform (EC 3.6.3.14) (V-ATPase A subunit 1)	−1.7
GRP78_MOUSE (P20029)	78 kDa glucose-regulated protein precursor (GRP 78) (Immunoglobulin heavy chain binding protein)	−1.7
Q4VWZ5 (Q4VWZ5)	Diazepam binding inhibitor, splice form 1b	−1.74
DEST_MOUSE (Q9R0P5)	Destrin (Actin-depolymerizing factor) (ADF) (Sid 23)	−1.75
HS90B_MOUSE (P11499)	Heat shock protein HSP 90-beta (HSP 84) (Tumor-specific transplantation 84 kDa antigen) (TSTA)	−1.75
CLH_MOUSE (Q68FD5)	Clathrin heavy chain	−1.8
TBA6_MOUSE (P68373)	Tubulin alpha-6 chain (Alpha-tubulin 6) (Alpha-tubulin isotype M-alpha-6)	−1.8
TBA8_MOUSE (Q9JJZ2)	Tubulin alpha-8 chain (Alpha-tubulin 8)	−1.8
ERMP1_MOUSE (Q3UVK0)	Endoplasmic reticulum metallopeptidase 1	−1.88
GNAO2_MOUSE (P18873)	Guanine nucleotide-binding protein G(o), alpha subunit 2	−1.88
Q5XJF8 (Q5XJF8)	Tubulin, alpha 1	−2
Q9CRC1 (Q9CRC1)	Adult male testis cDNA, RIKEN full-length enriched library, clone:4933425L11 product:fructose-bisphosphate aldolase A	−2.12
PGK2_MOUSE (P09041)	Phosphoglycerate kinase, testis specific (EC 2.7.2.3)	−2.23
MK01_MOUSE (P63085)	Mitogen-activated protein kinase 1 (EC 2.7.1.37) (Extracellular signal-regulated kinase 2) (ERK-2)	−2.27
SPTA2_MOUSE (P16546)	Spectrin alpha chain, brain (Spectrin, non-erythroid alpha chain) (Alpha-II spectrin)	−2.4
Q3TBV8 (Q3TBV8)	NOD-derived CD11c +ve dendritic cells cDNA, RIKEN full-length enriched library: pyruvate kinase	−2.4
HS90A_MOUSE (P07901)	Heat shock protein HSP 90-alpha (HSP 86) (Tumor-specific transplantation 86 kDa antigen) (TSTA)	−2.67
EF1G_MOUSE (Q9D8N0)	Elongation factor 1-gamma (EF-1-gamma) (eEF-1B gamma)	−2.7
ACON_MOUSE (Q99KI0)	Aconitate hydratase, mitochondrial precursor (EC 4.2.1.3) (Citrate hydro-lyase) (Aconitase)	−2.73
DHPR_MOUSE (Q8BVI4)	Dihydropteridine reductase (EC 1.5.1.34) (HDHPR) (Quinoid dihydropteridine reductase)	−2.98
DPYL2_MOUSE (O08553)	Dihydropyrimidinase-related protein 2 (DRP-2) (ULIP 2 protein)	−3.5
Q5SQX7 (Q5SQX7)	Cytoplasmic FMR1 interacting protein 2 (Fragment)	−4.5
GDIC_MOUSE (Q61598)	Rab GDP dissociation inhibitor beta-2 (Rab GDI beta-2) (GDI-3)	−4.76
Q8VCE0 (Q8VCE0)	Na+/K+-ATPase alpha 3 subunit	−5.5

**Table 3:** Four-way Venn diagram analysis of GO functional group creation by up/down regulated protein sets from cortex and hippocampus of 3xTgAD mice.

**a**	**b**
Cell maturation	Clathrin-coated endocytosis
Chromatin assembly	Cu^2+^ binding
Creatine kinase activity	Calmodulin binding
Cytochrome c reductase activity	Di/tri-valent cation transport
Negative regulation of microtubule polymerization	Lipid binding
Mg^2+^/Cu^2+^ binding	Metal ion homeostasis
Heterotrimeric G protein	Neuron projection
ER-Golgi transport	
DNA fragmentation	
Dopamine metabolism	
Cytochrome c reductase activity	
Creatine kinase activity	
Chromatin assembly	
Cell maturation	
Apoptosis	
Neurotransmitter uptake	
SOD removal	
Synaptosome structure	
Syntaxin binding	
Vesicle docking exocytosis	
**c**	**d**
Iron/calcium binding	Acetyl coA catabolism
NADH dehydrogenase activity	GTPase activity
Neurotransmitter transport	GTP binding
SNARE binding	Hydrolyase activity
Actin binding	Na^+^/K^+^ ATPase activity
Regulation of actin filament depolymerization	Protein tyrosine kinase activity
Anti-oxidant activity	Protein folding/polymerization
Clathrin-coated vesicle	Translation elongation factor activity
Cell development	Actin cytoskeleton
Exocytosis	Dendrite morphology
Learning-memory	Response to heat/stress
Lipid binding	Microtubule-based movement
Mitochondrial electron transport	
Negative regulation of cell organization	
Neuron development	
Neuron differentiation	
Neurogenesis	
Transmission of nerve impulse	
Oxidative phosphorylation	
Regulation of synapse plasticity	
Synaptic vesicle-mediated transport	
**f**	**g**
ATP-synthesis-coupled proton transport	Cell-cell signaling
Neurotransmitter secretion	Regulation of neurotransmitter levels
Synaptic transmission	
**h**	
K^+^ transport	
**i**	
Glycolysis	
Neurite morphology	

Specific loci are identified alphabetically according to Figure 8.

**Table 4:** Four-way Venn diagram analysis of significantly regulated KEGG signaling pathway creation by up/down regulated protein sets from cortex and hippocampus of 3xTgAD mice.

**a**	**b**
Adherens junction	Ca^2+^ signaling
Tight junction	Glyoxylate metabolism
Urea cycle	Long term potentiation (LTP)
Cell communication	
**c**	**d**
Amyotrophic lateral sclerosis	Antigen processing/presentation
Parkinson's disease	Axon guidance
Phenylalanine metabolism	Type II diabetes
Aspartate metabolism	TCA cycle
Glutamate metabolism	
**f**	**g**
Alzheimer's disease	SNARE interactions
Neurodegenerative disorders	
**h**	**i**
Focal adhesions	Insulin signaling
Gap junctions	
Long term depression (LTD)	
MAPK signaling	
Regulation of actin cytoskeleton	
**k**	**l**
Huntington's disease	Glycolysis
**m**	**o**
ATP synthesis	Oxidative phosphorylation
Carbon fixation	

Specific loci are identified alphabetically according to Figure 11.

### Co- and contra-regulated protein expression levels in the hippocampus and cortex

**Figure 2:**
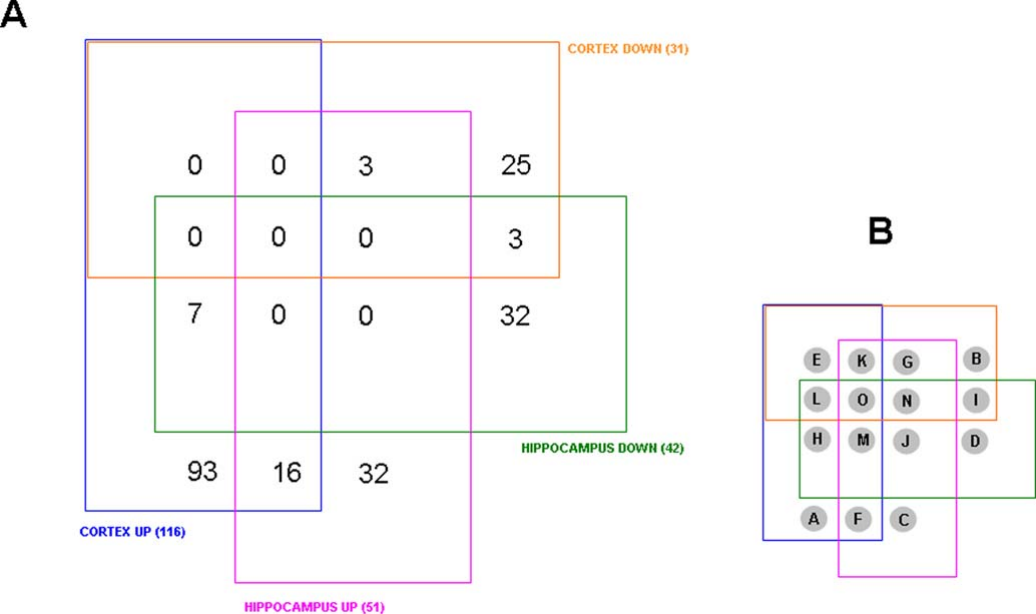
Four-way Venn diagram analysis for significantly regulated proteins in 3xTgAD compared to control from cortex and hippocampus. A. Numbers of proteins existing in the 15 loci in the Venn diagram between the four paradigms, *i.e.* up- or down-regulation in cortex or hippocampus. B. Key for loci in the four-way Venn diagram in A.

**Figure 3:**
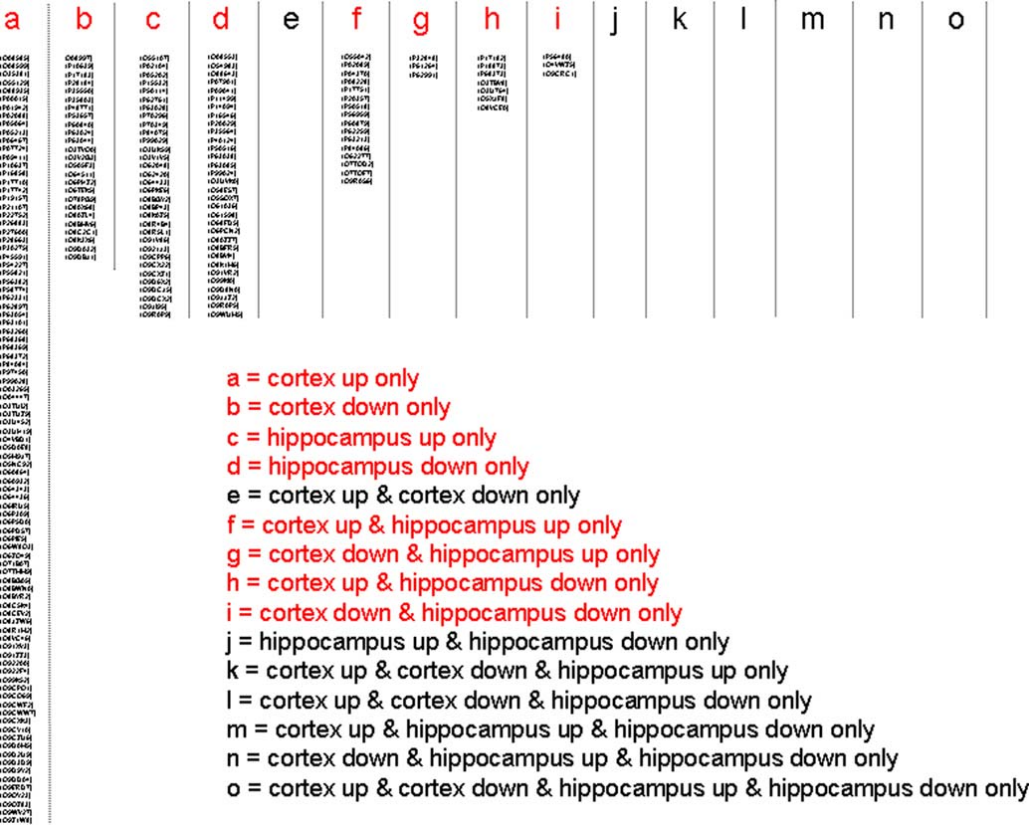
Murine protein accession ID numbers from four-way Venn diagram analysis and loci identification. The fifteen possible loci from a four-way Venn diagram are listed in the alphabetical key from Figure 2. Loci that contain occupied (accession numbers) protein sets are indicated in red and also in the descriptor lines for the loci.

**Figure 4:**
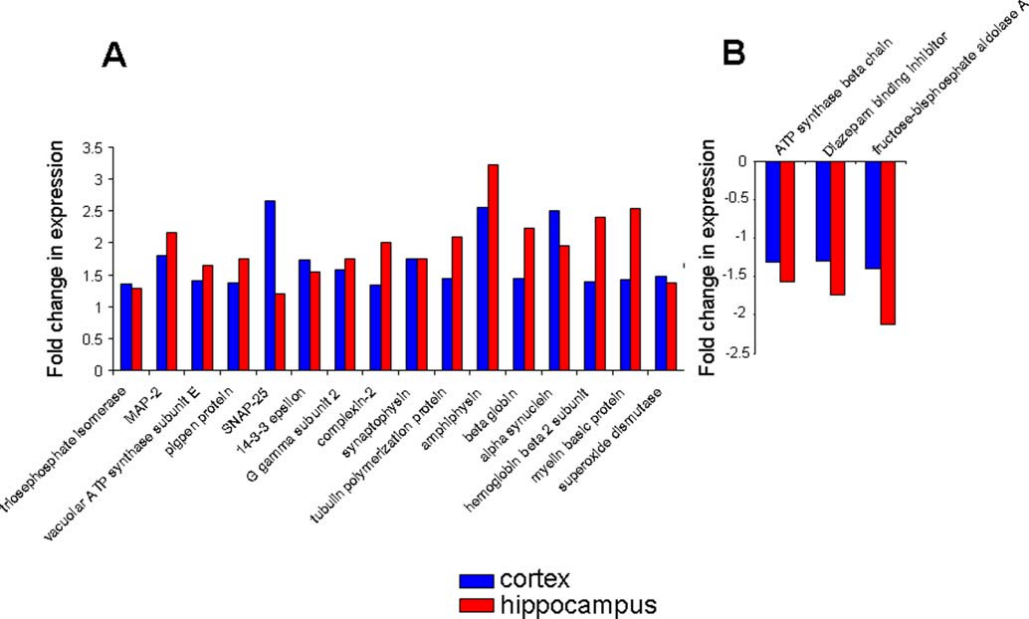
Co-regulated proteins between cortex and hippocampus in 3xTgAD mice compared to control. A. Proteins that were elevated in their expression in 3xTgAD mice compared to control in both the cortex (blue) and hippocampus (red). B. Co-regulated proteins in 3xTgAD animals that were diminished compared to control animals.

The numerous proteins identified from the hippocampus and cortex and their expression level relative to control animals, were arranged according to their expression and relative expression levels in a four-way Venn diagram (Fig. 2: http://www.pangloss.com/seidel/Protocols/venn.cgi). We found that there was a good specificity between proteins regulated (either up or down) in the cortex and in the hippocampus, *i.e.* no proteins were both up-regulated and down-regulated in both the tissues. Additionally, there was a greater similarity between the cortex and hippocampus with respect to protein up-regulation as there were 16 proteins up-regulated in both the cortex and hippocampus. In contrast, there were only three proteins that were co-downregulated in both the cortex and hippocampus. Similarly, when the diverse regulation of protein expression was investigated, there were more proteins (seven) that were up-regulated in the cortex and down-regulated in the hippocampus, compared to three proteins down-regulated in the cortex and up-regulated in the hippocampus. The multiple expression and tissue combinations from the four-way Venn diagram and the protein IDs are listed in Fig. 3. In Fig. 4 the proteins co-regulated between the cortex and hippocampus in AD animals compared to the controls are shown. It is interesting to note that many of the co-upregulated proteins are involved in synaptic neurotransmission (SNAP-25, complexin, synaptophysin, alpha-synuclein), energy management (triosephosphate isomerase, superoxide dismutase) and cytoskeletal dynamics (tubulin polymerization protein). The co-downregulated proteins were fewer in number, but also seemed to be more selectively involved with energy balance (*e.g*. ATP synthase, fructose bisphosphate aldolase A). This could suggest that in the 3xTgAD animals there was an increased attempt to maintain neuronal energy balance and increase neurotransmitter secretion. In addition to these energy metabolism proteins, there was also a concerted reduction of the diazepam binding inhibitor protein (DBI).

**Figure 5:**
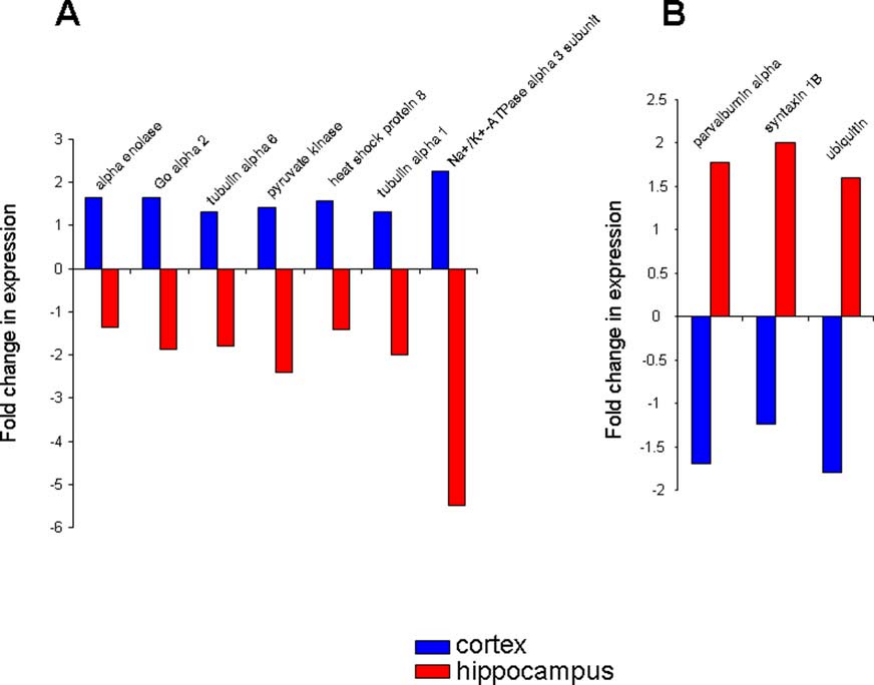
Contra-regulated proteins between cortex and hippocampus in 3xTgAD mice compared to control. A. Proteins that were potentiated in 3xTgAD compared to control in the cortex and attenuated in the hippocampus of 3xTgAD compared to control. B. Proteins that were potentiated in 3xTgAD compared to control in the hippocampus and attenuated in the cortex of 3xTgAD compared to control.

Among the contra-regulated proteins between the hippocampus and cortex (Fig. 5), some of the predominant phenotypes included structural/stress proteins (tubulin, heat-shock protein 8) as well as energy-related factors such as pyruvate kinase and the Na^+^/K^+^ ATPase alpha 3 subunit, which was selectively up-regulated in the cortex, while down-regulated in the hippocampus. The contra-regulation of the heterotrimeric G_α_o G protein is interesting as this G protein links many receptor signaling systems to ion channels involved in maintaining neuronal excitability, *e.g*. the inward rectifying potassium channels. With respect to the converse scenario (down-regulated in cortex and up-regulated in hippocampus) the proteins in this specific subset are involved in maintaining neuronal survival (parvalbumin), neurotransmission (syntaxin) and protein processing (ubiquitin).

**Figure 6:**
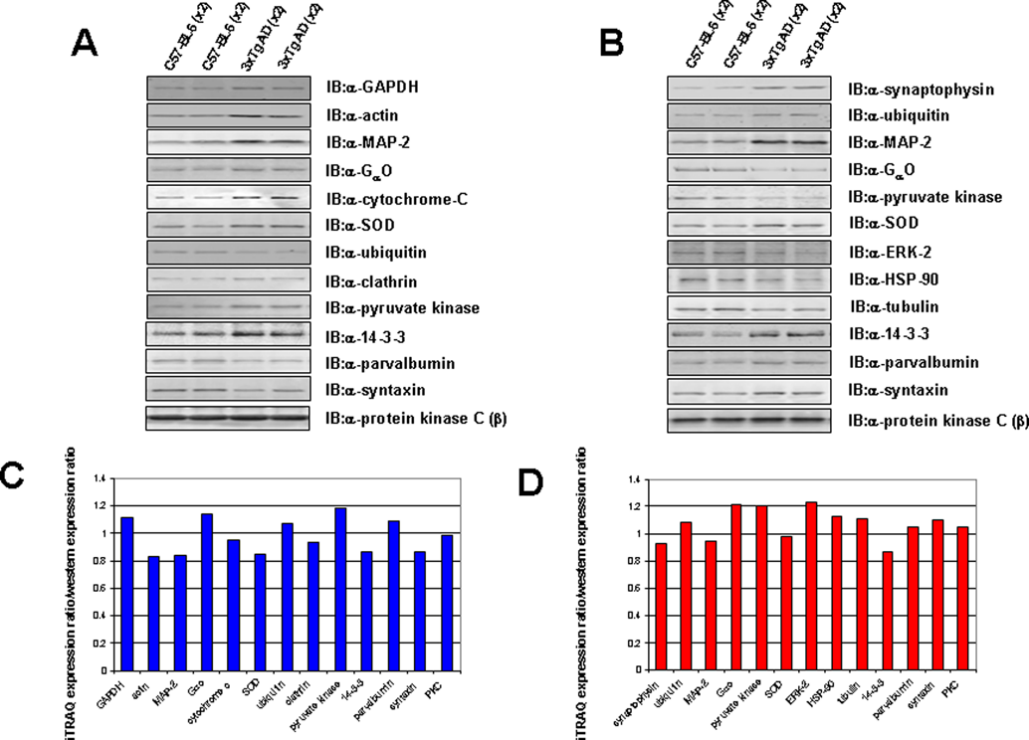
Western blot analysis of a selected array of proteins significantly regulated in the cortex or hippocampus of 3xTgAD mice compared to control. A. Representative western blots of loaded cortex protein resolved by SDS-PAGE and specifically western blotted with the antisera indicated. Lanes 1 and 2 contain protein from the control cortices and lanes 3 and 4 from the cortices of 3xTgAD animals. B. Western blots from protein extracted from hippocampal samples. The column format of the panel follows an identical format to panel A. C. Histogram depicting the similarity between measured cortex protein iTRAQ expression ratios and the western blot expression ratios of 3xTgAD to wild-type. Mathematical division of cortex iTRAQ expression ratios for multiple proteins by the western blot expression ratios yields values all near unity indicating a strong agreement in expression data. D. Histogram demonstrates a similar (to panel C) close relationship between iTRAQ and western blot expression ratios for proteins from the hippocampus. As each value for multiple proteins is near unity there is a strong agreement between iTRAQ expression and western blot expression analysis for hippocampal proteins as well as cortical proteins.

To verify the expression of a broad selection of proteins co- or contra-regulated by AD in the hippocampus or cortex, western blots were performed from tissue lysate samples resolved onto PVDF membranes (Fig. 6). We verified the proteomic data that we obtained using the iTRAQ technique by performing western blot analyses for a total of 16 proteins that were significantly altered in either the hippocampal or cortical tissues (compared to control) and a protein (protein kinase C) that did not show significant differences between wild-type and 3xTgAD tissues. The western blot validation of these multiple proteins reinforced the power of multi-protein analyses of complex samples such as hippocampal and cortical tissue from AD animal models (Figure 6). To demonstrate the relationships between the iTRAQ expression ratios gained and the expression ratios observed for the same proteins with western blots, the following mathematical procedure was performed. The iTRAQ expression ratio for each protein, in cortex or hippocampus, was divided by the mean ratio (of the two 3xTgAD samples compared to the two control samples) of western blot expression ratio. Each of the protein values obtained were all similar to unity (0.08>iTRAQ ratio/western ratio<1.2; Figure 6) indicating a consistency of expression profile between iTRAQ and western blot data.

### Phenotypic functional group analysis of complex protein alterations in AD brain compared to control brain

**Figure 7:**
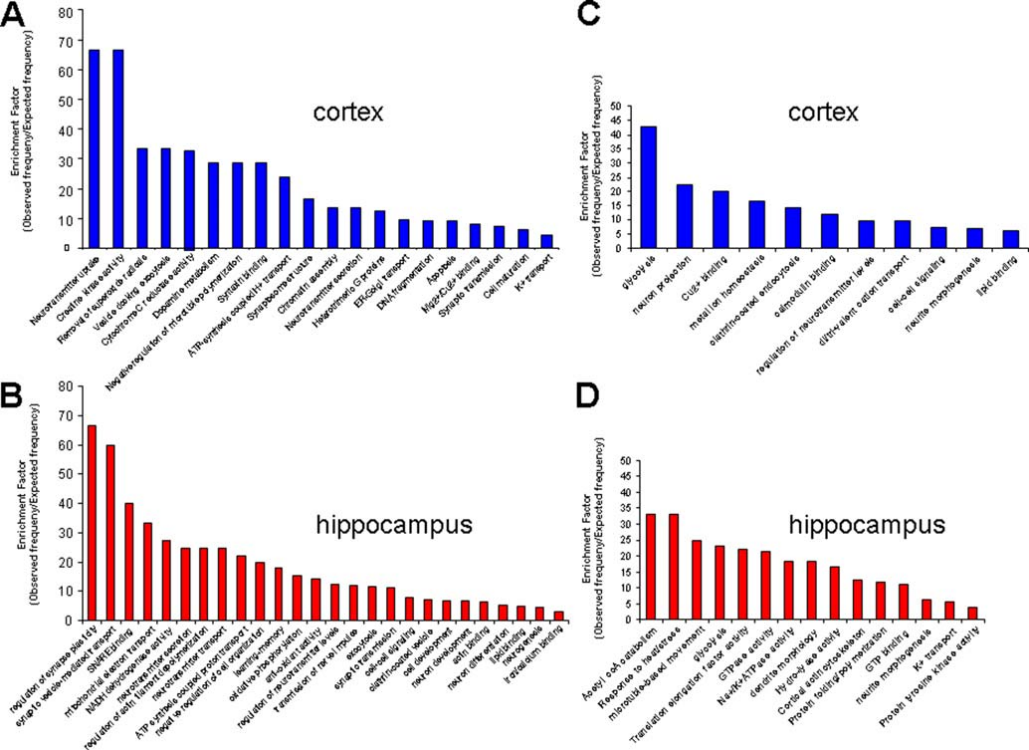
Gene-ontology (GO) functional group analysis of up- or down-regulated protein sets from cortex and hippocampus from 3xTgAD animals. Panels A (cortex) and B (hippocampus) depict the significantly represented (p<0.05) functional GO groups and their relative enrichment factor compared to background murine sets created by the up-regulated proteins (in 3xTgAD compared to control) identified in each tissue. Panels C (cortex) and D (hippocampus) depict analogous data to Panels A and B but for input protein sets down-regulated in 3xTgAD compared to control.

**Figure 8:**
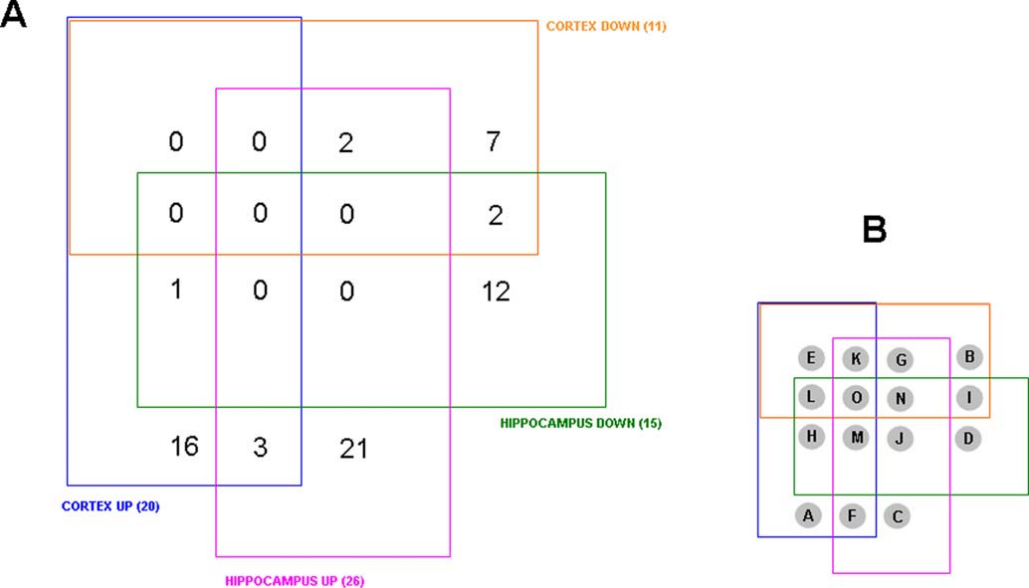
Four-way Venn diagram analysis for significantly regulated GO functional groups in 3xTgAD compared to control from cortex and hippocampus. A. Numbers of functional groups existing in the potential 15 loci in the Venn diagram between the four paradigms, *i.e*. up- or down-regulation in cortex or hippocampus. B. Key for loci in the four-way Venn diagram in A. The functional groups represented in the occupied loci are listed in Table 3.

**Figure 9:**
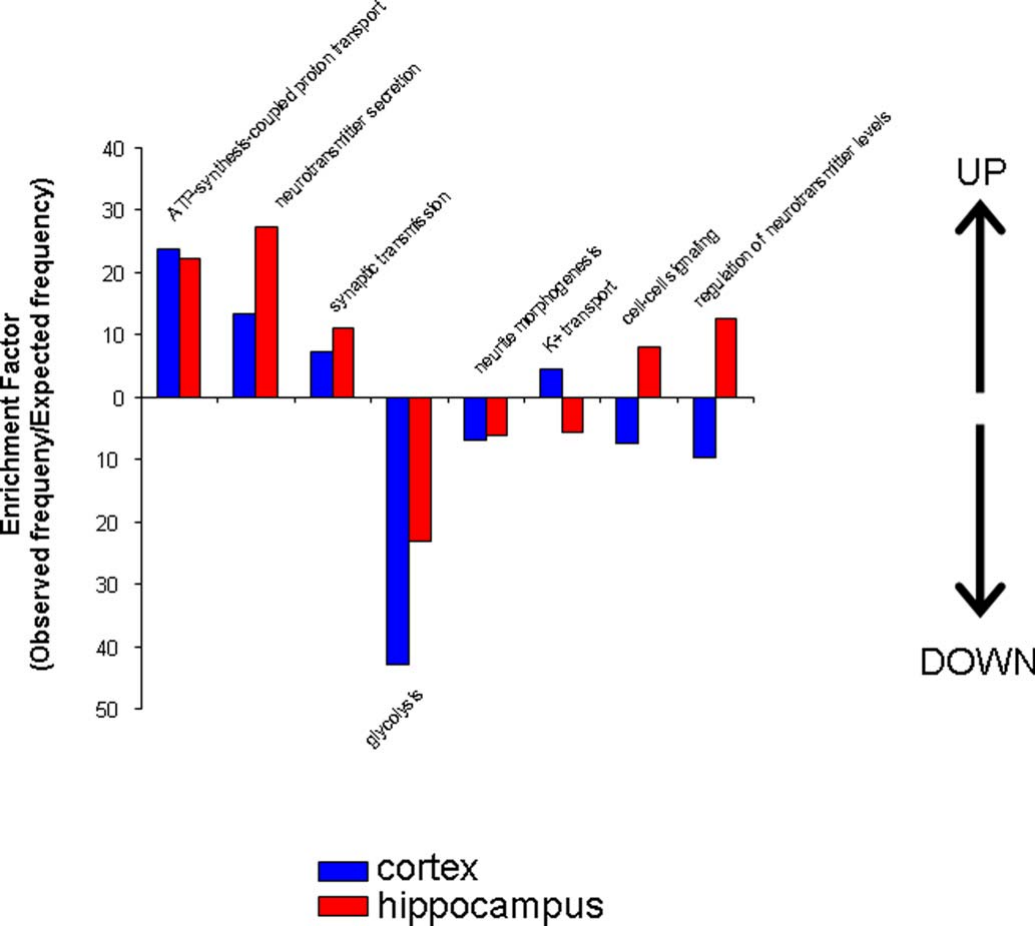
Co- and contra-regulated GO functional groups created by up- and down- regulated protein sets from cortex and hippocampus. Functional group enrichment factors for up-regulated functional groups (designated with UP arrow) from cortex and hippocampus are shown alongside enrichment factors for down-regulated protein groups (designated with DOWN arrow).

To utilize the wide range of data obtained on protein alterations, we used a gene set analysis toolkit, WebGestalt ([18]: http://bioinfo.vanderbilt.edu/webgestalt/) in which protein identifications were input using a Swiss-Prot ID for the proteins identified. This toolkit allows the functional annotation of gene/protein sets into gene-ontology (GO) functional groups and also into well characterized functional signaling pathways (KEGG: Kyoto Encyclopedia of Genes and Genomes, http://www.genome.jp/kegg/). Not only is functional annotation possible, but an enrichment score can also be obtained of the frequency of occurrence of a specific protein (or gene) within any given experimental subset with respect to a species-specific background set. Thus, an enrichment factor (observed frequency in input set/expected expression frequency in background species set) can be created that has a statistically testable value, indicating that the protein (or gene) is specifically over- or under-represented in the input sample group. When allotting proteins into functional groups (GO) or physiological signaling pathways (KEGG), at least two proteins were required to be independently placed into that functional group before using any statistical analysis. The significance of the representation of functional groups or pathways in the AD samples versus control was assessed using a built-in hypergeometric test with a p<0.05 cut-off. Input protein lists of up- or down-regulated proteins were analyzed using the Webgestalt software. The up-regulated and down-regulated proteins from cortex or hippocampus once submitted to the pathway algorithms, created the significantly (p<0.05) represented functional groups shown in Fig. 7. To analyze correlations between the functional groups significantly up- or down-regulated in 3xTgAD mice versus controls, a four-way Venn diagram was constructed. In this Venn diagram (Fig. 8.), the majority of functional groups were singly represented in each of the tissue or regulation direction paradigms. There were three significantly up-regulated groups shared between the cortex and hippocampus (ATP-synthesis-coupled proton transport, neurotransmitter secretion, synaptic transmission; Fig. 9). In contrast, there were two significantly over-represented pathways down-regulated in both cortex and hippocampus (glycolysis, neurite morphology; Fig. 9). There was one contra-regulated functional group (potassium transport) which was up-regulated in the cortex while down-regulated in the hippocampus, and there were two contra-regulated functional groups (cell-cell signaling, regulation of neurotransmitter levels) which were down-regulated in the cortex but up-regulated in the hippocampus.

### Phenotypic physiological signaling pathway analysis of complex protein alterations in 3xTgAD versus control mice

**Figure 10:**
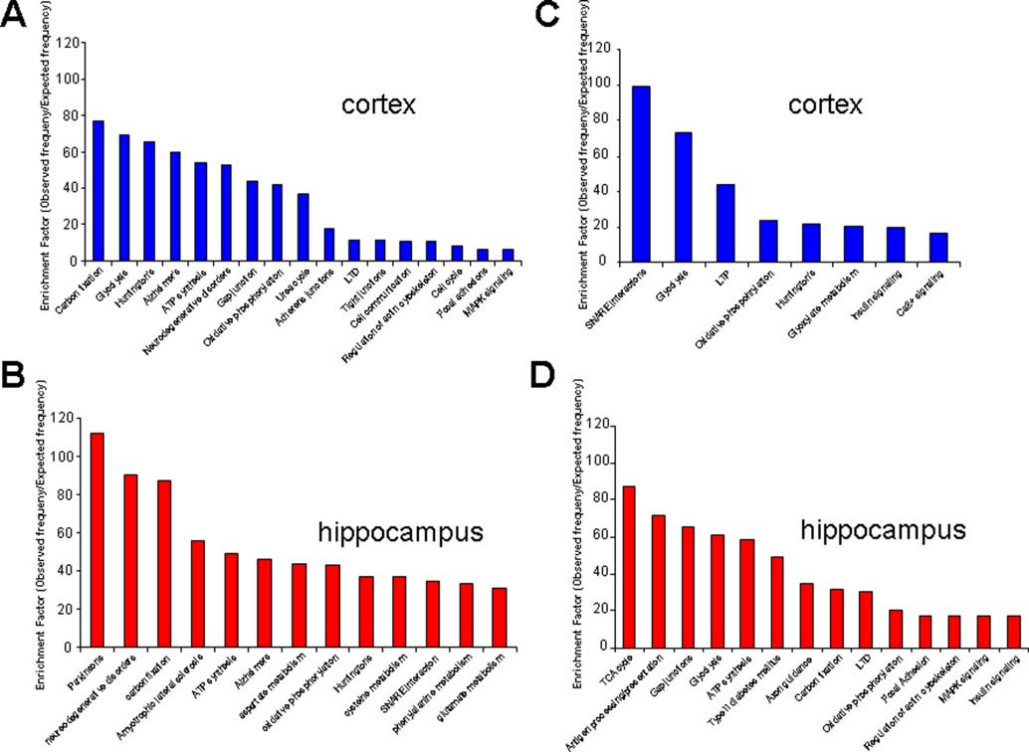
KEGG signaling pathway analysis of up- or down-regulated protein sets from cortex and hippocampus from 3xTgAD animals. Panels A (cortex) and B (hippocampus) depict the significantly represented (p<0.05) KEGG signaling pathways and their relative enrichment factor compared to background murine sets created by the up-regulated proteins (in 3xTgAD compared to control) identified in each tissue. Panels C (cortex) and D (hippocampus) depict analogous data to Panels A and B but for input protein sets down-regulated in 3xTgAD compared to control.

**Figure 11:**
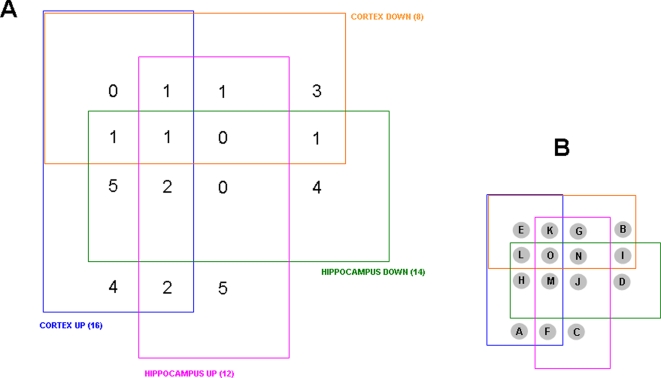
Four-way Venn diagram analysis for significantly regulated KEGG signaling pathways in 3xTgAD compared to control from cortex and hippocampus. A. Numbers of functional groups existing in the potential 15 loci in the Venn diagram between the four paradigms, *i.e*. up- or down-regulation in cortex or hippocampus. B. Key for loci in the four-way Venn diagram in A. The functional groups represented in the occupied loci are listed in Table 4.

**Figure 12:**
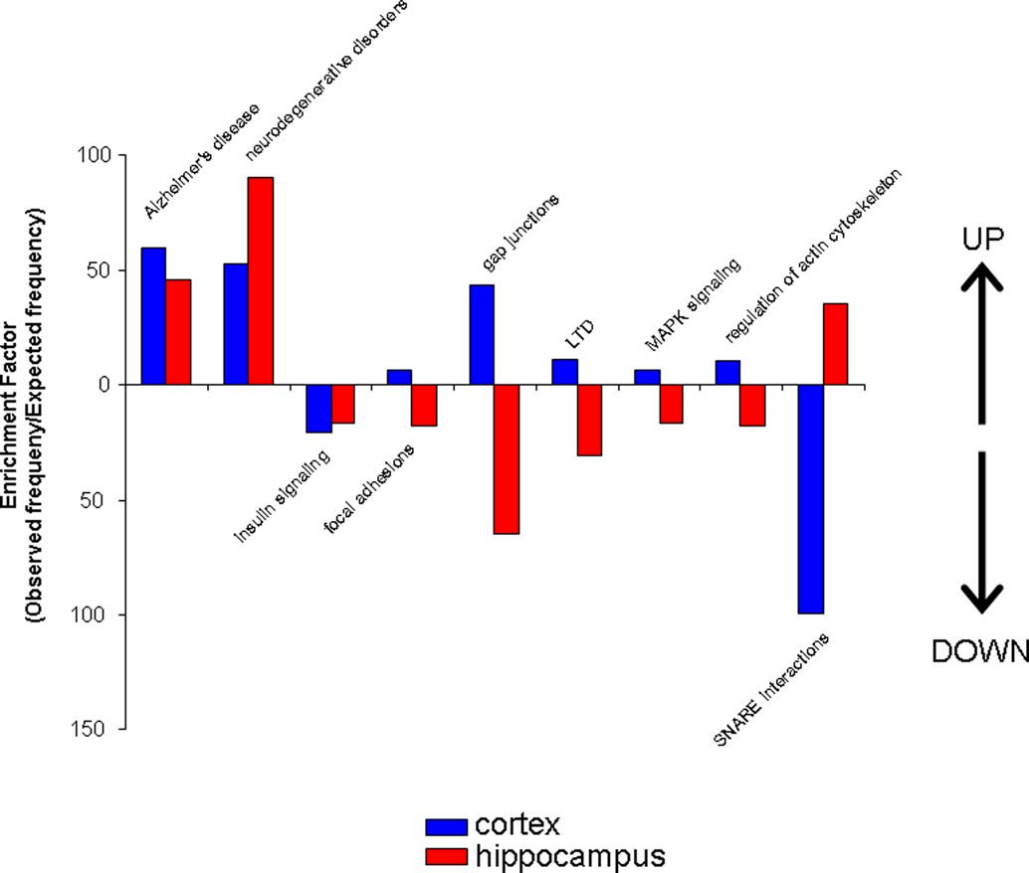
Co- and contra-regulated KEGG signaling pathways created by up- and down-regulated protein sets from cortex and hippocampus. Functional group enrichment factors for up-regulated (designated with UP arrow) from cortex and hippocampus are shown alongside enrichment factors for down-regulated protein groups (designated with DOWN arrow).

In a similar manner to the functional group classifications using GO terms, the up- and down-regulated protein sets from the cortex and hippocampus were also analyzed using the KEGG pathway algorithm. From the analysis of the individual proteins, to the functional GO group analysis, to this KEGG pathway analysis, there is an ascending complexity of functional grouping. However, with this widening of functionality there is also a greater capacity for cross-over and inclusion of seemingly contradictory protein expression effects as complex signaling pathways can encompass diverse regulatory proteins. The significantly overrepresented KEGG pathways in all four tissue and protein expression regulation paradigms are shown in Fig. 10. Similarly to the GO term analysis, we created a four-way Venn diagram to cross-analyze relationships between the two central nervous tissues studied (Fig. 11). As expected, there was a complicated relationship between the different tissues and the up- or down-regulation of the protein groups. Considering the nature of the sample and the complex unbiased analysis performed, it is interesting to note that the two significantly co-upregulated (cortex and hippocampus) signaling pathways include the Alzheimer's disease pathway and the neurodegenerative disorders pathway (Fig. 12). The presence of these two pathways confirms the validity of mass protein analytical techniques combined with unbiased multiprotein annotation in predicting functional groups from complex tissue samples. Interestingly with respect to maintenance of neuronal energy balance, the only co-regulated pathway from the down- regulated protein groups in cortex and hippocampus was the insulin signaling pathway. A large cluster of functional signaling pathways were up-regulated in the cortex and down-regulated in the hippocampal samples, *i.e*. focal adhesions, gap junctions, long-term depression (LTD), mitogen-activated/microtubule-associoated protein kinase (MAPK) signaling and regulation of actin cytoskeleton. In contrast, only one signaling pathway was represented significantly in the set of cortex down-regulated and hippocampus up-regulated proteins, *i.e*. SNARE interactions.

## Discussion

Neurological disorders such as AD are inherently complex in that they involve the disruption of perhaps the most intricate entity in existence, the neuronal network in the human brain. The changes that occur in AD at the cellular and molecular levels undoubtedly include both pathophysiological degenerative processes and adaptive responses to counteract the disease process. In this study we used an iTRAQ proteomic approach to elucidate molecular alterations that may occur during the clinical course of AD. We analyzed multiple protein expression patterns in the cortex and hippocampus of 16 month-old 3xTgAD mice compared to their age-matched controls. At this age, the 3xTgAD mice exhibit considerable amyloid and tau pathology in the hippocampal and cortical regions and they have learning and memory impairments [17].

Quantitative protein expression data was obtained from four groups of two pooled individuals for the cortex and the hippocampus. As there is considerable discussion about the relative merits of pooling tissues before analysis or pooling data post individual analysis, we therefore decided to take a hybrid approach. Our approach consisted of partial pooling of tissues. Two animals were pooled (control (114) or 3xTgAD (116)) for a single isobaric label but then another, completely separate, two-animal pool was used for a second control (115) or 3xTgAD (117) isobaric label set. While pooling of animal tissue does not specifically reduce any inter-individual variation, it can however dilute the differential expression effects between individuals. It is clearly preferable to perform parallel investigations upon individuals in a population sample and then to pool the individual data sets, as this preserves any idiosyncrasies between animals or subjects that can then be correlated later with specific expression profiles. The only drawback for this analysis though is one of cost to the investigator. Typically, tissue pooling is preferred when either small amounts of tissue are available from individuals or high numbers of individuals are used in the study. One of the primary arguments supporting the practice of pooling biological samples for large scale protein or gene analysis is the notion that biological variation can be reduced by this practice [19]. However, pooling can attenuate the effects of biological variation but not of course the intrinsic cause of the biological variation itself [20]. The basic assumption underlying sample pooling is that of biological averaging, *i.e.* the measure of interest taken on the pool of samples is equal to the average of the same measure taken on each of the individual samples which contributed to the pool [21]. It has been argued that even when biological averaging does not hold, pooling can be of use and inferences concerning differential gene or protein expression may not be adversely affected by tissue pooling [20]. The significant alterations in proteins that were detected using our hybrid method demonstrates that with this mixture of biological averaging and parallel analysis, differential expression can be assessed from small animal numbers. Employment of unbiased parametric analysis of complex protein sets, as we have used in this study, may greatly enhance the utility of mass spectrometry as a tool to understand physiological changes occurring in disease. Unbiased clustering of proteins into functional groups (gene ontology) and especially pathways (KEGG) allows a physiological ‘phenotype’ of the protein set to be created that is not entirely dependent on specific protein identities in the set. Hence with pathway analysis, variations in output protein identifications (even in biological replicates) will be compensated for by perhaps other proteins in the sample that also can fall into and significantly populate a specific pathway group. The nature of the output pathway phenotype, indicative of the biology of the input sample, therefore may be more reproducible (even between multiple mass spectrometry runs) as the parametric analysis uses multiple protein identities to create a significantly regulated group rather than using a single identity.

For the cortex, a total of 142 significantly regulated proteins were identified (116 up-regulated, 26 down-regulated). For the hippocampus a total of 91 significantly regulated proteins were identified (49 up-regulated, 42 down-regulated). In many cases, the proteins, and their direction of regulation were specific to either the cortex or hippocampus. However, there were several discrete subsets of co-regulated proteins and contra-regulated proteins, and these protein sets are expected to hold more significance as they represent specific commonalities and distinctions in how different brain regions respond to a genetically-imposed pathological state.

The higher levels of superoxide dismutase (SOD) in the cortex and hippocampus of the 3xTgAD mice compared to controls, is consistent with previous data suggesting that oxidative stress is increased in association with amyloid and neurofibrillary pathologies in AD patients and in mouse models of AD [15], [19], [20]. Additionally, the increased amount of glyceraldehyde-3-phosphate dehydrogenase (GAPDH) in the cortex of 3xTgAD mice compared to controls, could reflect the activation of neurodegenerative pathways, as recent findings have suggested that GAPDH can promote neuronal death [21].

We identified distinct sub-groups of proteins with common functional actions that were co-upregulated in both the hippocampus and cortex, in part due to or perhaps in response to, the presence of the AD pathology. For example, we identified proteins that are involved in synaptic transmission and vesicle exocytosis such as SNAP-25, complexin-2, synaptophysin, amphiphysin and alpha synuclein. It is interesting and perhaps revealing that these up-regulations may seem paradoxical in light of many reports of down-regulation of synaptic proteins in AD; however many of these reports are from post-mortem tissue collected from human patients with severe end-stage dementia [22]. One explanation for the increase in synaptic proteins in 3xTgAD mice is that neurons respond to the neuropathology in an attempt to ameliorate synaptic transmission deficits. Similar modest increases in synaptophysin and SNAP-25 have also been observed in other animal models of AD [23]. The increased levels of alpha-synuclein in the brain samples from 3xTgAD mice are consistent with the reported accumulation of alpha-synuclein in the brains of AD patients [24]. Interestingly, our finding of a paradoxical increase in amphiphysin levels also seems consistent with AD pathology, as Kelly and Ferreira [25] recently demonstrated increases in hippocampal neuronal amphiphysin in response to an Aβ load. In addition to the group of proteins related to synaptic function, our iTRAQ analysis identified another important cluster of proteins involved in cytoskeletal dynamics, including MAP-2 and tubulin polymerization promoting protein (TPPP). MAP-2 may accumulate in the neurons of 3xTgAD mice through its capacity to stabilize itself and accumulate in neurofibrillary tangles [26]. TPPP has recently been demonstrated (using electron microscopy) to form an integral part of neurofibrillary tangles in AD and Lewy body-related disorders [27]. The scaffolding adapter 14-3-3 proteins may also be related to the structural subset of up-regulated proteins in both the cortex and hippocampus. Indeed, using immunohistochemistry it has been demonstrated that there are significant correlations of 14-3-3 reactivity with elevated levels of neurofibrillary tangles in AD [28]. Additionally, we identified pigpen which is a nuclear protein with an RNA-binding motif and a putative transcriptional activation domain [29] and is considered to be a critical controller of transcriptional regulation in response to trophic growth factors resulting in cell proliferation/differentiation [30]. While evidence for a role of pigpen protein in the pathology of AD is presently lacking, our demonstration of increased pigpen levels in 3xTgAD brain tissue, suggests a role for this protein in cellular responses to AD pathology.

With respect to the proteins involved in energy balance, there seems to be a multi-faceted effect of the AD process in cortical and hippocampal cells. Hence, some metabolism-related proteins were co-upregulated (triosephosphate isomerase and vacuolar ATP synthase subunit E), while others were co-downregulated (ATP synthase beta chain and fructose-bisphosphate aldolase A) in the cortex versus the hippocampus of the 3xTgAD mice. This potential *yin and yang* of metabolic balance appears to flow through many levels of the unbiased analysis of proteins whose expression is significantly altered in AD pathology. The other primary co-downregulated protein identified between the cortex and hippocampus was the diazepam binding inhibitor protein (DBI). DBI was first isolated from rat and human brain, and is found almost exclusively in GABAergic neurons where it is believed to inhibit GABAergic neurotransmission [31]. DBI has been proposed to block the binding of endogenous benzodiazepines (endozepines) to the regulatory site present on the GABA_A_ receptor, thus reducing the regulation of the chloride channel current. Several reports have also linked DBI to neurological disorders including AD, Parkinson's disease (PD) and schizophrenia [32]. Additionally, in human hippocampal samples, an AD-related diminution (greater than that observed in Parkinson's disease) of the expression of DBI has been demonstrated [33]. The functional correlates of this reduction in DBI could be an adaptive response to the disruption of synaptic strength which has been proposed to occur in AD, *i.e*. inhibition of inhibitory signaling may serve to enhance an ailing neurotransmitter system. Of course it is facile to also predict that this effect could be a mediator of the neuropathologies of AD, *i.e*. a decrease in inhibitory neurotransmission would also facilitate a potential increase in excitotoxic neurotransmission events. This potential duality of factors in a complex system such as the CNS reinforces the need for a higher level of understanding of multiple correlated factors (facilitated by quantitative proteomics) when attempting to illuminate a complex disease process such as AD.

In contrast to the co-regulated proteins, fewer contra-regulated proteins were observed between the cortex and hippocampus; however, their identity suggests a differential activity status between these two tissues in AD. Amongst proteins up-regulated in the cortex, a structural bias is evident in that tubulins and heat shock proteins were selectively up-regulated in the cortex while they were down-regulated in the hippocampus; additionally, energy-related proteins (enolase, pyruvate kinase and Na^+^/K^+^-ATPase subunits) were a small sub-group of these proteins as well. This suggests that either the hippocampus does not require an up-regulation of metabolic proteins or that the cortex is undergoing a greater energy deficit in the 3xTgAD mice and thus requires a more significant modification of its intermediary cell metabolism. To further understand the molecular underpinning of AD, it is useful to consider the contra-regulated proteins that were elevated in the hippocampus but down-regulated in the cortex. One of these contra-regulated proteins, parvalbumin, is a cytoplasmic calcium binding protein that buffers intracellular Ca^2+^, reducing the activity of Ca^2+^-dependent potassium channels, and thus altering calcium-dependent neuronal firing patterns [34]. Parvalbumin is primarily found in a subset of fast-spiking, inhibitory GABAergic interneurons. Interestingly, parvalbumin-containing neurons are very resistant to neurotoxic damage from ischemia [35], epilepsy [36] and overt NMDA receptor stimulation [37]. Syntaxins are a group of synaptic plasma membrane proteins that were first demonstrated to physically interact with PS-1 by Smith et al. [38] and were subsequently shown, *in vitro*, to alter APP processing via this interaction with PS-1, resulting in an attenuation of secreted Aβ [39]. It is also interesting to note that in the hippocampus there is a specific up-regulation of ubiquitin, perhaps in an attempt to attenuate the deleterious effects of Aβ and hyperphosphorylated tau upon the ubiquitin-proteasome system (UPS: [40]). Taken together, these three proteins and their relative regulation in the hippocampus, relative to the cortex, suggests that perhaps the hippocampus is being preferentially protected, potentially at the expense of cortical tissue. In this view, the metabolic differences between the hippocampus and cortex occur due to an excessive strain put on the cortex due to a preferential rescue of hippocampal tissue.

When the proteins that were altered similarly in the cortex and hippocampus of 3xTgAD mice compared to control mice were analyzed according to functional groups (using Gene Ontology terms), three significantly up-regulated protein sets were identified-ATP-synthesis-coupled proton transport, neurotransmitter secretion and synaptic transmission. From many lines of experimental evidence it is clear that global energy management is crucial to neuronal survival during times of stress and disease, and therefore it is not surprising that proton transport links both tissues in our study. Many anti-oxidant factors that play a neuroprotective role in AD also aid in proton transport, including coenzyme Q-10 [41], [42]. The functional groups of proteins that were present at lower levels in the cortex and hippocampus of the 3xTgAD mice consisted of proteins involved in glycolysis and a group of proteins involved in neurite morphogenesis. The magnitude of the functional group enrichment in the cortical samples is considerably greater than that for the hippocampus (created by more proteins being present in the cortical rather than the hippocampal sample that cluster into this functional group), which again suggests that the hippocampus is being preferentially protected during the disease process. The general reduction in neurite morphogenesis is presumably indicative of the considerable changes in the neural networks, which may be related to impaired cognitive function in the 3xTgAD mice [17].

Among the contra-regulated groups, two were present where there was hippocampal up-regulation and cortical down-regulation (cell-cell signaling, and regulation of neurotransmitter levels) while only one functional group occurred with cortical up-regulation and hippocampal down-regulation (K^+^ transport). As with the inferences from primary protein identification, the classification into functional groups also hints at a hippocampally-biased adaptive response, in that there is a more profound control of neurotransmitter levels. Again whether this is indicative of a heightened pathological load and thus a greater response to it or a conserved protective mechanism to maintain short-term memory remains to be conclusively determined.

When considering the potential signaling pathways that are controlled by the multiple changes in protein expression that we have described, it is reassuring to know that a coherent demonstration of the accuracy of the protein identification was achieved. Hence, we observed that the two co-upregulated KEGG pathways in cortex and hippocampus were AD and neurodegenerative disorders. The significant presentation of these two KEGG pathways reinforces the potential utility of this mass protein identification and quantification technique coupled to an un-biased functional annotation algorithm. In addition to the expected alterations, novel signaling pathways also seemed to be altered in the 3xTgAD mice, including insulin signaling which suggests an alteration in trophic/metabolic regulation at the level of the CNS. A substantial subset of contra-regulated KEGG pathways were demonstrated that showed up-regulation in the cortex and down-regulation in the hippocampus (gap junctions, long-term depression [LTD], MAPK signaling, and regulation of the actin cytoskeleton). The role of gap junction activity in the pathophysiology of AD is presently unclear [43]; however, increased expression of gap junction proteins such as connexin-43 have been reported at the sites of amyloid plaques [44]. Also of interest is the SNARE interaction pathway which was up-regulated in the hippocampus, but not in the cortex, of the 3xTgAD mice. Due to the larger breadth of proteins included in signaling pathways it is more likely that complex regulations of the same pathway could occur in the same tissues. For example there are several cases of signaling pathways that are both up- and down-regulated in the same tissue, *e.g*. ATP synthesis is both up- and down-regulated in the hippocampus, while the broad pathway of proteins related to oxidative phosphorylation is up- and down-regulated in both the cortex and hippocampus. These crossovers of function may seem paradoxical, but could be indicative of a disturbed cellular metabolism caused by the pathological alterations (*i.e*., Aβ and tau pathology) in the neurons of the 3xTgAD mice.

From this study we have gained a greater appreciation of the intricacies of the interaction between a multi-faceted disease process and a complex neuronal system. In assessing the presence of pathophysiology one must appreciate that the tissue or organ that is diseased is not merely a passive entity and that its cells may react in a specific manner to the imposed insult. Therefore complicated signaling pathways and functional groups of proteins may show both positive and negative regulation to create the resultant phenotype. Because the 3xTgAD mice exhibit Aβ deposits, tau pathology, synaptic dysfunction, spatial learning deficits and anxiety-like behaviors [17], [45], [46], the proteins and pathways identified as being altered in the hippocampus and cortex of the 3xTgAD mice may either contribute to the synaptic dysfunction and behavioral abnormalities, or they could contribute to neuroprotective responses of the neurons. By using proteomic techniques, such as iTRAQ, to gain a deeper understanding of the complex signaling pathways and functional groups of proteins that are altered in AD, we could potentially open up novel therapeutic avenues for the treatment of AD.

## Materials and Methods

### Tissue and protein extraction

The cortex and hippocampus were carefully dissected out from 8 male 16 month-old C57/BL6 control mice and 8 male 16 month-old 3xTgAD mice. All procedures were performed in accordance with approved institutional protocols and were approved by the Institutional Animal Care and Use Committee of the National Institute on Aging. The tissues were homogenized on ice with an NP-40-based cell lysis solution as described previously [47], and subsequently the tissue was incubated at 4°C for 1 hour with constant agitation. The lysate was then clarified by centrifugation at 14,000 rpm for 15 minutes at 4°C. The total protein concentration of the clarified lysate was measured and all the samples were normalized to the same protein concentration (1 mg/ml). A total of 50 µg of cortex or hippocampus protein extract from each animal (control or 3xTgAD) was then pooled for each isobaric iTRAQ (Applied Biosystems) mass tag label, *i.e*. 114, 115, 116 or 117. Thus, for the cortical 114 mass tag labeling reaction, 50 µg of protein from control mouse 1 was added to 50 µg of protein from control mouse 2 (Figure S1). The same was then performed for the hippocampal samples. Each mass tag labeling reaction was performed using 100 µg of total trypsinized protein, obtained from two animals. Before labeling, and for the preservation of the proteins, the total protein was precipitated into a pellet using acidification with 30% tricholoroacetic acid.

### iTRAQ

Protocol-iTRAQ chemistry labeling reagents were obtained from Applied Biosystems. Control and 3xTgAD tissue samples were treated in parallel throughout the labeling procedure. The generic labeling protocol consisted of the following steps: protein reduction and cysteine blocking, digestion of proteins with trypsin, labeling of peptides with iTRAQ reagents, combining the samples that were to be compared, strong cation exchange chromatography, desalting with solid phase extraction, and LC/MS/MS analysis. Briefly, protein sample pellets (generated by trichloroacetic acid precipitation) were dissolved in dissolution buffer (0.5 M triethyammonium bicarbonate, TEAB), to give a 5 µg/µl concentration. Subsequently, 20 µl (100 µg) of each mixture was aliquoted and 1 µl denaturant was added. Reducing reagent (2 µl) was added and the tubes were incubated at 60°C for 1 hour. The proprietary (methyl methane-thiosulphonate, Applied Biosystems) cysteine blocking reagent (1 µl) was added and incubated for another 10 minutes at room temperature. Trypsin (Promega) was reconstituted in water, 10 µl of the solution containing 10 µg of trypsin was added and incubated overnight at 37°C. Before labeling, the reagents were dissolved in ethanol and the contents of one vial were transferred to a sample tube. The labeling took place for 1 h at room temperature. The following labels were used for cortical samples: 114-control (C1)-2 pooled C57/BL6 animals; 115-control (C2)-2 pooled C57/BL6 animals; 116-3xTgAD (C3)-2 pooled 3xTgAD animals; 117-3xTgAD (C4)-2 pooled 3xTgAD animals. The following labels were used for hippocampus samples: 114-control (H1)–2 pooled C57/BL6 animals; 115-control (H2)-2 pooled C57/BL6 animals; 116-3xTgAD (H3)–2 pooled 3xTgAD animals; 117-3xTgAD (H4)-2 pooled 3xTgAD animals. After labeling, the sample tubes, for control and AD for separate tissues, were combined and dried down to a volume of 50 µl to reduce the content of ethanol prior to strong cation exchange (SCX) chromatography.

### Western blot analysis

20 µg samples of the cortex or hippocampus were mixed with a denaturing and reducing Laemmli buffer [47] and resolved by SDS-PAGE. The proteins were then electrotransferred from the gel onto a polyvinylenedifluoride (PVDF: NEN Life Sciences) screen. The screen was treated with a Tris-buffered saline solution supplemented with Tween-20, NP-40 and 4% bovine serum albumin to block non-specific antibody interactions. Primary antibodies (1∶500–1000) were applied to the PVDF membrane for 1 hour at room temperature and proteins were identified by the application of an alkaline-phosphatase-conjugated secondary antibody (1∶10000) that recognized the species type of the primary antibody. The PVDF membrane was then exposed to an enzyme-linked chemifluorescent developing substrate (Amersham Biosciences) and was scanned using a GE Typhoon 9410 phosphorimager at a resolution of 100 µm. Western blot images were then quantified using GE ImageQuant 5.1 software. The primary antibodies were obtained from the following sources: ERK2, tubulin, G_α_O, actin, 14-3-3-ε, MAP-2, clathrin, parvalbumin-α, HSP-90, syntaxin, GAPDH and protein kinase C were obtained from Santa Cruz; ubiquitin, SOD, pyruvate kinase were obtained from AbCam; cytochrome C was obtained from Cell Signaling Technology; synaptophysin antibodies were obtained from Sigma.

### Strong Cation Exchange Chromatography

SCX chromatography was employed to separate and resolve labeled peptides as well as to remove excess reagents, MS interfering compounds and undigested proteins. The SCX column (75 µm×10 cm: particles -C-18-AQ, 5 µm:120 Å: YMC) was equilibrated with loading buffer (10 mM potassium phosphate in 25% acetonitrile, pH 3.0). The iTRAQ-labeled sample was diluted 10x with loading buffer and then loaded on a PolyLC PolySULFOETHYL A column. Subsequently, 200 µL, containing approximately 100 µg of peptides, was loaded onto the column. Peptides were eluted at 50 µl/min in 40 min gradient, using solvent A (10 mM potassium phosphate in 25% acetonitrile, pH 3.0) and solvent B (350 mM KCl in 10 mM potassium phosphate in 25% acetonitrile, pH 3.0). Absorbance at 280 nm was monitored and ten 2 minute fractions were collected for LCMS/MS analysis (Figures S2 and S3). The microflow HPLC conditions employed for the cation exchange were applied using an Agilent 1100 series capillary LC. Samples (3 µl) were passed through a peptide trap into the LC column. A gradient was run between 0.1% formic acid (A) and 90% acetonitrile: 0.1% formic acid (B) as follows, 5% B to 40% B over 60 minutes. The flow rate was 20 µl/min and was then split to 200 nl/min.

### Mass Spectrometry analysis

An Applied Biosystems QStar mass spectrometer was used for the isolation and collision-induced dissociation of input peptides. The electrospray voltage typically maintained was 2.5kV. Mass spectrometer calibration was performed with a mixture of CsI (MW 132.9049), synthetic peptide ALILTLVS (829.5393) and verapamil (455.2904). The general conditions for mass ion identification and isobaric mass tag resolution were: Scan Events: 1: Survey 400–1200 Da; 2–4: Data dependent MS/MS on 3 most intense ions from 1. For collision energy: rolling collision energy (automatically set according to the m/z of precursor), increased for 20% due to iTRAQ tags. The exclusion time used for analysis was 60 seconds. Mass tolerance was set to 0.15 atomic mass units for precursor and 0.1 atomic mass units for fragmented ions. Data analysis was performed using the ProQuant program suit (Applied Biosystems). Protein identification was performed using the most recently updated murine SwissProt database. Raw peptide identification results were processed to generate a minimal set of proteins, as previously described, using the Paragon Algorithm (Applied Biosystems) [51], [52]. Briefly, the raw peptide identification results from the Paragon Algorithm searches were further processed by the ProGroup Algorithm (Applied Biosystems) within the Protein Pilot software before final display. The ProGroup Algorithm uses the peptide identification results to determine the minimal set of proteins that can be reported for a given protein confidence threshold. For each protein ProGroup reports two score types for each protein: *unused* ProtScore and *total* ProtScore. The *total* ProtScore is a measurement of all the peptide evidence for a protein (analogous to commonly reported protein scores). The *unused* ProtScore is a measurement of all the peptide evidence for a protein that is not better explained by a higher ranking protein. Hence *unused* ProtScore is calculated by using the unique peptides that are not linked to a higher ranking protein. The protein confidence threshold cutoff for this study was ProtScore 2.0 (*unused*) with at least two peptides with a 95% confidence. Proteins identified with mass tag changes (ratio >1.2 or <0.8) that were consistent between two independent biological experiments were manually validated and quantified by two independent analysts. These arbitrary cutoffs for expression variation have been implemented by multiple researchers [51]–[55]. Peak areas for each of the signature ions (114, 115, 116, 117) were obtained and corrected according to the manufacturers' instructions to account for isotopic overlap. Only those signature ions with intensities less than 1500 counts were used for quantitation, greater than 1500 counts results in detector saturation.

To calculate the relative protein levels, proteins with a statistically significant label ratio of 116/117:114 greater or equal to 1.2 were considered to be proteins elevated in 3xTgAD versus control samples. Proteins with a significant label ratio of 116/117:114 less than or equal to 0.8 were indicative of down-regulated proteins in AD versus control. The relative expression level for ‘up-regulated’ proteins was calculated as follows: the mean 116/117:114 label ratio was divided by the mean 115:114 label ratio. The eventual relative expression level of ‘down-regulated proteins’ was calculated as follows: the mean 115:114 label ratio was divided by the mean 116/117:114 label ratio and then made negative to indicate the relative direction of expression compared to control.

## Supporting Information

Figure S1 Coomassie staining of protein extracts for iTRAQ labeling. The extracted proteins (10 microgram samples per lane) from control (C57/BL6: animals 1–4) or 3xTgAD mice (animals 5–8) are shown in a coomassie-stained SDS-PAGE gel. The protein extracts from two pooled animal cortices or hippocampi were then used in the labeling reaction, denoted by the horizontal bars underneath. The masses of the molecular mass markers on the left of each gel represent, in descending order in kDa, 203, 119, 100, 51.9, 37.3, 29, 19.5 and 6.9.
(0.39 MB TIF)

Figure S2 SCX UV trace and LC-MS/MS base peak chromatograms for pooled samples of control or 3xTgAD cortex samples. A. SCX UV Chromatogram (using 280nm wavelength absorbance measurements of peptide bonds). Initial peak between 0–10 min contains SDS and other reagents from the iTRAQ labeling reaction. B. LC-MS/MS base peak chromatogram of fractions F1–F10 of the labeled and mixed cortex control/3xTgAD samples from A.
(0.08 MB TIF)

Figure S3 SCX UV trace and LC-MS/MS base peak chromatograms for pooled samples of control or 3xTgAD hippocampal samples. A. SCX UV Chromatogram (using 280nm wavelength absorbance measurements of peptide bonds). Initial peak between 0–10 min contains SDS and other reagents from the iTRAQ labeling reaction. B. LC-MS/MS base peak chromatogram of fractions F1–F10 of the labeled and mixed hippocampus control/3xTgAD samples from A.
(0.08 MB TIF)

## References

[1] Ashford JW (2004). APOE genotype effects on Alzheimer's disease onset and epidemiology.. J Mol Neurosci.

[2] Braak H, Braak E, Grundke-Iqbal I, Iqbal K (1986). Occurrence of neuropil threads in the senile human brain and in Alzheimer's disease: a third location of paired helical filaments outside of neurofibrillary tangles and neuritic plaques.. Neurosci Lett.

[3] Selkoe DJ (1991). The molecular pathology of Alzheimer's disease.. Neuron.

[4] Trojanowski JQ, Shin RW, Schmidt ML, Lee VM (1995). Relationship between plaques, tangles and dystrophic processes in Alzheimer's disease.. Neurobiol Aging.

[5] Masliah E (1995). The natural evolution of the neurodegenerative alterations in Alzheimer's disease.. Neurobiol Aging.

[6] Hashimoto M, Masliah E (2003). Cycles of aberrant synaptic sprouting and neurodegeneration in Alzheimer's and dementia with Lewy bodies.. Neurochem Res.

[7] Hyman B, Gomez-Isla T (1994). Alzheimer's disease is a laminar regional and neural system specific disease, not a global brain disease.. Neurobiol Aging.

[8] Augustinack JC, Schneider A, Mandelkow EM, Hyman BT (2002). Specific tau phosphorylation sites correlate with severity of neuronal cytopathology in Alzheimer's disease.. Acta Neuropathol.

[9] Braak H, Braak E (1996). Evolution of the neuropathology of Alzheimer's disease.. Acta Neurol Scand Suppl.

[10] Minoshima S, Foster NL, Kuhl DE (1994). Posterior cingulate cortex in Alzheimer's disease.. Lancet.

[11] Zola-Morgan S, Squire LR, Amaral DG (1989). Lesions of the hippocampal formation but not lesions of the fornix or the mammillary nuclei produce long-lasting memory impairment in monkeys.. J Neurosci.

[12] Dekosky ST, Scheff SW, Styren SD (1996). Structural correlates of cognition in dementia: quantification and assessment of synapse change.. Neurodegeneration.

[13] Scheff SW, Price DA (2003). Synaptic pathology in Alzheimer's disease: a review of ultrastructural studies.. Neurobiol Aging.

[14] Masliah E (2001). Recent advances in the understanding of the role of synaptic proteins in Alzheimer's disease and other neurodegenerative disorders.. J Alzheimer's Dis.

[15] Mattson MP (2004). Pathways towards and away from Alzheimer's disease.. Nature.

[16] Roberson ED, Mucke L (2006). 100 years and counting: prospects for defeating Alzheimer's disease.. Science.

[17] Oddo S, Caccamo A, Shepherd JD, Murphy MP, Golde TE (2003). Triple-transgenic model of Alzheimer's disease with plaques and tangles: intracellular Abeta and synaptic dysfunction.. Neuron.

[18] Zhang B, Kirov S, Snoddy J (2005). WebGestalt: an integrated system for exploring gene sets in various biological contexts.. Nucleic Acids Res.

[19] Churchill GA, Oliver B (2001). Sex, flies and microarrays.. Nat Genet.

[20] Kendziorski C, Irizarry RA, Chen KS, Haaj JD, Gould MN (2005). On the utility of pooling biological samples in microarray experiments.. Proc Natl Acad Sci USA.

[21] Zhang SD, Gant TW (2004). A statistical framework for the design of microarray experiments and effective detection of differential gene expression.. Bioinformatics.

[22] Praticò D, Uryu K, Leight S, Trojanoswki JQ, Lee VM (2001). Increased lipid peroxidation precedes amyloid plaque formation in an animal model of Alzheimer amyloidosis.. J Neurosci.

[23] Butterfield DA, Sultana R (2007). Redox proteomics identification of oxidatively modified brain proteins in Alzheimer's disease and mild cognitive impairment: insights into the progression of this dementing disorder.. J Alzheimers Dis.

[24] Hara MR, Agrawal N, Kim SF, Cascio MB, Fujimuro M (2005). S-nitrosylated GAPDH initiates apoptotic cell death by nuclear translocation following Siah1 binding.. Nat Cell Biol.

[25] Minger SL, Honer WG, Esiri MM, McDonald B, Keene J (2001). Synaptic pathology in prefrontal cortex is present only with severe dementia in Alzheimer disease.. J Neuropathol Exp Neurol.

[26] Bailey JA, Lahiri DK (2006). Neuronal differentiation is accompanied by increased levels of SNAP-25 protein in fetal rat primary cortical neurons: implications in neuronal plasticity and Alzheimer's disease.. Ann NY Acad Sci.

[27] Attems J, Quass M, Jellinger KA (2007). Tau and alpha-synuclein brainstem pathology in Alzheimer disease: relation with extrapyramidal signs.. Acta Neuropathol.

[28] Kelly BL, Ferreira A (2007). Beta-amyloid disrupted synaptic vesicle endocytosis in cultured hippocampal neurons.. Neuroscience.

[29] Zhang EY, DeTure MA, Bubb MR, Caviston TL, Erdos GW (1996). Self-assembly of the brain MAP-2 microtubule biding region into polymeric structures resembling Alzheimer filaments.. Biochem Biophys Res Commun.

[30] Kovacs GG, Laszlo L, Kovacs J, Jensen PH, Lindersson E (2004). Natively unfolded tubulin polymerization promoting protein TPPP/p25 is a common marker of alpha-synucleinopathies.. Neurobiol Dis.

[31] Sugimori K, Kobayashi K, Kitmura T, Sudo S, Koshino Y (2007). 14-3-3 protein beta isoform is associated with 3-repeat tau neurofibrillary tangles in Alzheimer's disease.. Psychiatry Clin Neurosci.

[32] Alliegro MC (2000). A C-terminal carbohydrate-binding domain in the endothelial cell regulatory protein, pigpen: new function for an EWS family member.. Exp Cell Res.

[33] Allapatt SR, Zhang M, Zhao X, Alliegro MA, Alliegro (2003). Mouse pigpen encodes a nuclear protein whose expression is developmentally regulated during craniofacial morphogenesis.. Dev Dyn.

[34] Barbaccia ML, Costa E, Ferrero P, Guidotti A, Roy A (1986). Diazepam-binding inhibitor. A brain neuropeptide present in human spinal fluid: studies in depression, schizophrenia and Alzheimer's disease.. Arch Gen Psychiatry.

[35] Ferrero P, Benna P, Costa P, Tarenzi L, Baggio G (1988). Diazepam binding inhibitor-like immunoreactivity (DBI-LI) in human CS. Correlations with neurological disorders.. J Neurol Sci.

[36] Edgar PF, Schonberger SJ, Dean B, Faull RL, Kydd R (1999). A comparative proteome analysis of hippocampal tissue from schizophrenic and Alzheimer's disease individuals.. Mol Psychiatry.

[37] Cello MR (1986). Parvalbumin in most gamma-aminobutyric acid-containing neurons of the rat cerebral cortex.. Science.

[38] Leifer D, Kowall NW (1993). Immunohistochemical patterns of selective cellular vulnerability in human cerebral ischemia.. J Neurol Sci.

[39] Sloviter RS, Sollas AL, Barbaro NM, Laxer KD (1991). Calcium-biding protein (calbindin-D28k) and parvalbumin immunocytochemistry in the normal and epileptic human hippocampus.. J Comp Neurol.

[40] Waldvogel HJ, Faull RLM, Williams NM, Dragunon M (1991). Differential sensitivity of calbindin/parvalbumin immunoreactive cells in the striatum to excitotoxins.. Brain Res.

[41] Smith SK, Anderson HA, Yu G, Robertson AG, Allen SJ (2000). Identification of syntaxin 1A as a novel binding protein for presenilin-1.. Brain Res. Mol. Brain Res..

[42] Suga K, Tomiyama T, Mori H, Akagawa K (2004). Syntaxin 5 interacts with presenilin holoproteins, but not with their N- or C-terminal fragments and affects beta-amyloid peptide production.. Biochem J.

[43] Upadhya SC, Hedge AN (2007). Role of ubiquitin proteasome system in Alzheimer's disease.. BMC Biochemistry.

[44] Dhanasekaran M, Ren J (2005). The emerging role of co-enzyme Q-10 in aging, neurodegeneration, cardiovascular disease, cancer and diabetes mellitus.. Curr Neurovasc Res.

[45] Beal MF (2004). Mitochondrial dysfunction and oxidative damage in Alzheimer's and Parkinson's disease and coenzyme Q-10 as a potential treatment.. J Bioenerg Biomembr.

[46] Nakase T, Naus CCG (2004). Gap junctions and neurological disorders of the central nervous system.. Biochimica et Biophysica Acta.

[47] Nagy JI, Li W, Hertzberg EL, Marotta CA (1996). Elevated connexin-43 immunoreactivity in Alzheimer's disease.. Brain Res.

[48] Billings LM, Oddo S, Green KN, McGaugh JL, LaFerla FM (2005). Intraneuronal Abeta causes the onset of early Alzheimer's disease-related cognitive deficits in transgenic mice.. Neuron.

[49] Nelson RL, Guo Z, Halagappa VM, Pearson M, Gray AJ (2007). Prophylactic treatment with paroxetine ameliorates behavioral deficits and retards the development of amyloid and tau pathologies in 3xTgAD mice.. Exp Neurol.

[50] Maudsley S, Pierce KL, Zamah AM, Miller WE, Ahn S (2000). The beta(2)-adrenergic receptor mediates extracellular signal-regulated kinase activation via assembly of a multi-receptor complex with the epidermal growth factor receptor.. J Biol Chem.

[51] Guo Y, Singleton PA, Rowshan A, Gucek M, Cole RN (2007). Quantitative proteomic analysis of human endothelial cell membrane rafts: Evidence of MARCKS and MRP regulation in the sphingosine 1-phosphate-induced barrier enhancement.. Mol Cell Proteomics.

[52] Donowitz M, Singh S, Salahuddin FF, Hogema BM (2007). Proteome of murine jejunal brush border membrane vesicles.. J Proteome Res.

[53] Griffiths SD, Burthem J, Unwin RD, Holyoake TL, Melo JV (2007). The use of isobaric tag peptide labeling (iTRAQ) and mass spectrometry to examine rare, primitive hematopoietic cells from patients with chronic myeloid leukemia.. Mol Biotechnol.

[54] Kassie F, Anderson LB, Higgins L, Pan Y, Matise I (2008). Chemopreventitive agents modulate expression profile of 4-(methylnitrosamino)-1(3-pyridyl)-1-butanone plus benzo[a]pyrene-induced lung tumors in A/J mice.. Carcinogenesis.

[55] Graham RL, Sharma MK, Ternan NG, Weatherly DB, Tarleton RL (2007). A semi-quantitative GeLC-MS analysis of temporal proteome expression in the emerging nosocomial pathogen Ochrobactrum anthropi.. Genome Biol.

