# Supplementary figures and images for: iTRAQ Analysis of Complex Proteome Alterations in 3xTgAD Alzheimer's Mice: Understanding the Interface between Physiology and Disease
Source: PLoS One. 2008 Jul 23;3(7):e2750. doi: 10.1371/journal.pone.0002750 (PMC2453232; doi:10.1371/journal.pone.0002750)

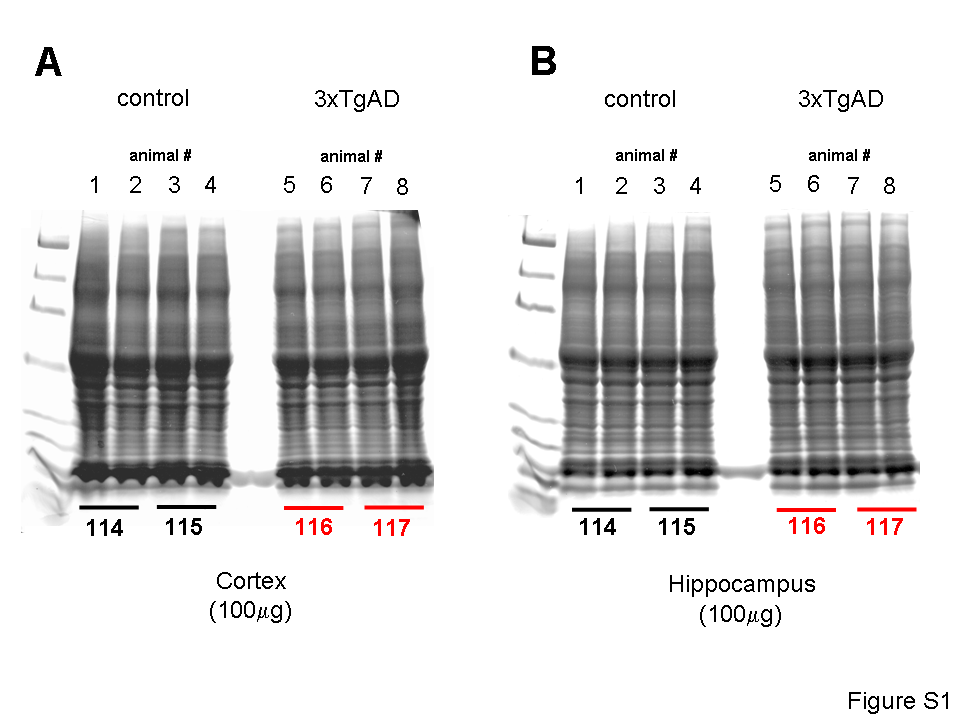

Supplement: Figure S1 — Coomassie staining of protein extracts for iTRAQ labeling. The extracted proteins (10 microgram samples per lane) from control (C57/BL6: animals 1–4) or 3xTgAD mice (animals 5–8) are shown in a coomassie-stained SDS-PAGE gel. The protein extracts from two pooled animal cortices or hippocampi were then used in the labeling reaction, denoted by the horizontal bars underneath. The masses of the molecular mass markers on the left of each gel represent, in descending order in kDa, 203, 119, 100, 51.9, 37.3, 29, 19.5 and 6.9. (0.39 MB TIF) [file pone.0002750.s001.tif]

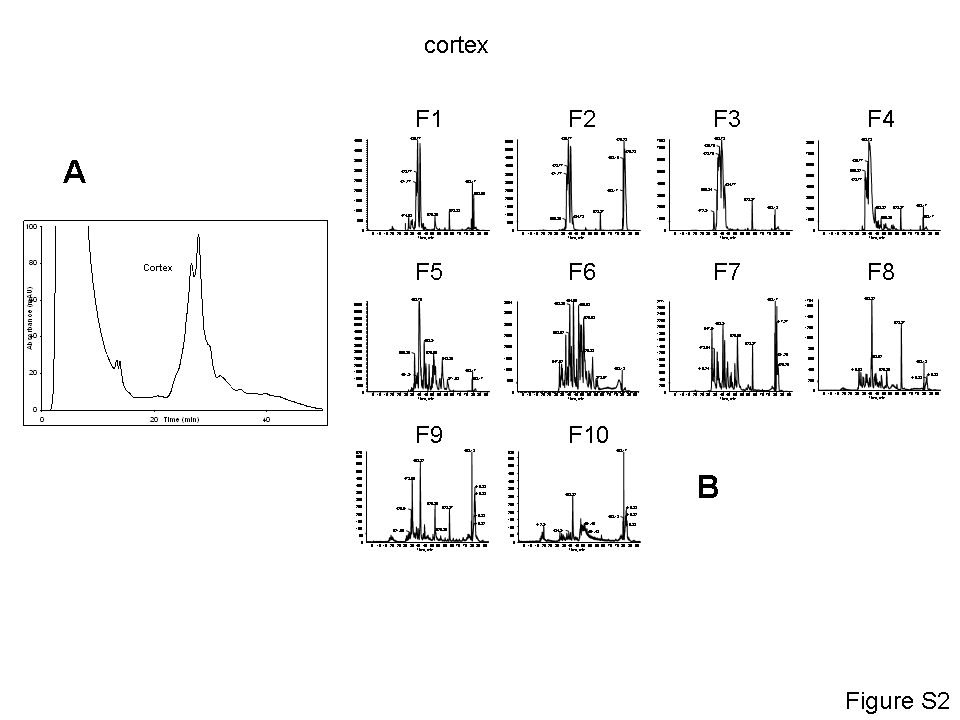

Supplement: Figure S2 — SCX UV trace and LC-MS/MS base peak chromatograms for pooled samples of control or 3xTgAD cortex samples. A. SCX UV Chromatogram (using 280nm wavelength absorbance measurements of peptide bonds). Initial peak between 0–10 min contains SDS and other reagents from the iTRAQ labeling reaction. B. LC-MS/MS base peak chromatogram of fractions F1–F10 of the labeled and mixed cortex control/3xTgAD samples from A. (0.08 MB TIF) [file pone.0002750.s002.tif]

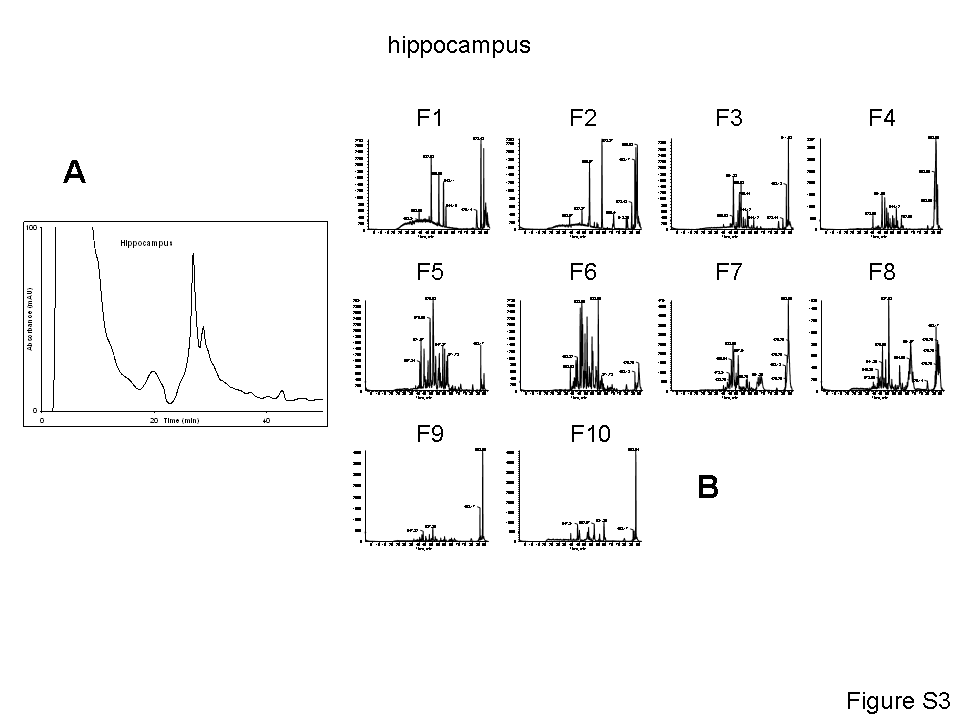

Supplement: Figure S3 — SCX UV trace and LC-MS/MS base peak chromatograms for pooled samples of control or 3xTgAD hippocampal samples. A. SCX UV Chromatogram (using 280nm wavelength absorbance measurements of peptide bonds). Initial peak between 0–10 min contains SDS and other reagents from the iTRAQ labeling reaction. B. LC-MS/MS base peak chromatogram of fractions F1–F10 of the labeled and mixed hippocampus control/3xTgAD samples from A. (0.08 MB TIF) [file pone.0002750.s003.tif]
